# A Molecularly Imprinted Polymer-Based Porous Silicon Optical Sensor for Quercetin Detection in Wines

**DOI:** 10.1021/acsami.4c21238

**Published:** 2025-02-11

**Authors:** Tiziano Di Giulio, Ibrar Muhammad Asif, Martina Corsi, Soumya Rajpal, Boris Mizaikoff, Nicoletta Ditaranto, Giuseppe E. De Benedetto, Cosimino Malitesta, Giuseppe Barillaro, Elisabetta Mazzotta

**Affiliations:** a Laboratory of Analytical Chemistry, Department of Biological and Environmental Sciences and Technologies (Di.S.Te.B.A.), University of Salento, via Monteroni, Lecce 73100, Italy; b Information Engineering Department, University of Pisa, via G. Caruso 16, Pisa 56122, Italy; c Institute of Analytical and Bioanalytical Chemistry, Ulm University, Albert-Einstein-Allee 11, Ulm 89081, Germany; d Hahn-Schickard, Sedanstrasse 14, 89077 Ulm, Germany; e Chemistry Department, Aldo Moro University of Bari, Via Orabona 4, Bari 70126, Italy; f Laboratory of Analytical Mass Spectrometry, Cultural Heritage Department, University of Salento, Via Monteroni, Lecce 73100, Italy

**Keywords:** molecularly imprinted polymer, vapor-phase polymerization, polymer simulations, porous silicon, quercetin detection, polypyrrole, wines analysis

## Abstract

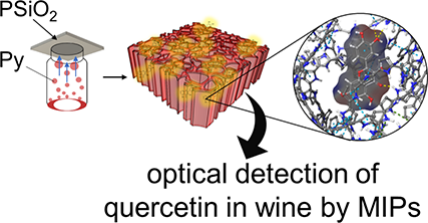

Quercetin (QU), a bioactive flavonoid with significant nutritional and antioxidant properties, plays a vital role in the quality and stability of wine. This study presents the development of a molecularly imprinted polymer (MIP)-based optical sensor for the selective and sensitive detection of quercetin in red and white wines. The sensor combines the selective molecular recognition capabilities of MIPs with the optical properties of nanostructured porous silica (PSiO_2_) scaffolds, which serve as the transducer. MIP synthesis was achieved through a novel room-temperature vapor-phase polymerization method using pyrrole as the functional monomer. Computational simulations were used to optimize pyrrole interactions with QU and at the polymer level, to explore the binding interactions of QU with the resulting polypyrrole (PPy) matrix. Comprehensive characterization including UV–vis reflectance spectroscopy and advanced surface analyses confirmed successful MIP formation. The sensor exhibited high sensitivity in a dual linear response range (2.5–80 μM and 80–200 μM), with a detection limit of 0.7 μM. Selectivity tests against structurally similar flavonoids and antioxidants demonstrated a significantly higher response to quercetin, with an imprinting factor of 3.6. The sensor was validated using real wine samples, demonstrating the ability to detect quercetin without prior sample preparation. Results showed strong agreement with high-performance liquid chromatography (HPLC), confirming the sensor reliability. Additionally, the sensor exhibited excellent reusability with minimal signal variation (RSD = 2.6%) and good stability over 60 days (RSD = 3%). This work highlights the potential of MIP-based optical sensors for the real-time monitoring of bioactive compounds in complex food matrices, such as wine, offering a robust and cost-effective alternative for quality control applications.

## Introduction

The global wine industry represents a multibillion-euro market, with producers and distributors operating worldwide.^1^ Rigorous control of wine properties is essential to enhance its sensory appeal, ensure nutritional integrity, and mitigate risks associated with raw materials and production processes.^2^ Chemically, wine is a complex mixture of organic acids, sugars, alcohols, esters, and polyphenols, particularly flavonoids.^3^ These compounds are influenced by grape variety, provenance, winemaking decisions during fermentation and aging, all of which affect wine quality and stability.^4^

Among these compounds, quercetin (QU) has garnered considerable attention due to its nutritional value and impact on winemaking.^5^ Quercetin is found in fruits and vegetables, grapes and wine.^5^ It is a potent antioxidant, with well-documented anti-inflammatory, antiallergic, anticancer, and antiviral properties.^6^ It also has a protective role in cardiovascular, neurological and respiratory health, as well as in preventing damage to skin tissues.^7^ Beyond its biological implications, quercetin plays a crucial role in wine sensory attributes, contributing to its astringency, bitterness, and overall flavor profile.^8^ However, high concentrations of quercetin can lead to sediment formation over time, potentially compromising the clarity and commercial viability of wine.^9^ Thus, monitoring quercetin levels is important for maintaining wine quality and ensuring desirable organoleptic properties.^7−9^

In this context, sensor-based approaches present a promising alternative to conventional analytical methods, which are often time-consuming, expensive, and require extensive sample preparation.^10^ Molecularly imprinted polymers (MIPs) have emerged as effective artificial receptors for sensors, offering high specificity, versatility, and robustness.^11−16^ MIPs are synthetic polymers designed with binding sites tailored to specific analytes, providing advantages over biological receptors, such as enhanced stability and compatibility with complex matrices.^17,18^

MIP-based sensors for QU have been assembled, mainly exploiting optical and electrochemical detection. For instance, Xu et al.,^19^ developed MOF-@MIP nanoparticles via free-radical precipitation polymerization for optical detection of QU in *Ginkgo biloba* extracts, achieving a linear range of 0–50 μM. However, low specificity evidenced by an imprinting factor of 1.8, was a limitation. Similarly, Hu et al.,^20^ developed Al(III)-coordinated imprinting polymer membranes on polypropylene fibers via thermally initiated radical polymerization, enabling a fluorescence-based detection up to 65 μM in spiked samples. Practical application was hindered by interference from structural analogues and labor-intensive sample preparation. Mantashloo et al.^21^ used sol–gel polymerization on graphene quantum dots for fluorescence quenching-based detection of QU, achieving an imprinting factor ∼4. However, significant pH sensitivity (∼70% signal loss under non-optimal conditions) restricted its applicability. Among electrochemical sensors, Ganjeh et al.,^22^ applied the electro-polymerization of l-cysteine on MXene/CuFe_2_O_4_-modified carbon paste electrodes, enabling the detection of QU within the range 0–10 μM. However, the sensor preparation required complex and time-consuming preparation of the carbon paste electrode before MIP electrodeposition. Bandyopadhyay et al.^23^ employed benzoyl peroxide-initiated thermal polymerization for reduced graphene oxide-based MIPs, enabling detection up to 400 μM, though the synthesis posed significant drawbacks. Sun et al.^24^ employed pyrrole electro-polymerization for QU detection but lacked detailed performance characterization, leaving issues like nonspecific adsorption and sensor stability unaddressed. In another work, Hurkul et al.^25^ developed a UV-photopolymerized sensor with pM-level sensitivity, but its limited range (0–10 pM) restricted broader applicability. These approaches highlight the need for rapid, and scalable approaches for MIP-based sensors suitable for real-time QU monitoring in complex matrices.

Recent advancements in polymerization techniques and functional monomers have broadened the integration of MIPs with various sensing platforms.^26,27^ However, incorporating MIPs into nanostructured transducers, which have the potential to enhance sensing performance through increased binding site density and reduced binding kinetics, remains challenging due to the difficulty of achieving a uniform thin MIP layer while preserving the nanoscale features of the transducer.^28^ Recently, a room-temperature vapor-phase polymerization method was reported enabling the deposition of a homogeneous MIP layer into nanostructured porous silicon (PSi) scaffolds for hemoglobin detection.^29^ PSi was used as the optical transducer^30−34^ for its customizable nanostructured features and cost-effective production.^35,36^ While nPSi is extensively used in (bio)sensing,^32−38^ its integration with MIPs remains largely unexplored.^29,37^

This work focuses on the development of an nPSi MIP-based optical sensor for quercetin detection in wine. Vapor-phase polymerization tailored to quercetin was used for the MIP incorporation within the nPSi transducer, using pyrrole as the functional monomer due to its ability to vaporize at room temperature.^29^ Computational calculations were employed to investigate pyrrole-quercetin interactions, revealing the versatility of pyrrole as a functional monomer capable of forming hydrogen bonds and π–π stacking interactions with quercetin, ensuring high selectivity and efficient imprinting. Computational studies at the polymer level further supported such results, enabling the evaluation of the binding interaction between QU and polypyrrole (PPy). The sensor detects quercetin in aqueous and ethanolic solutions across a concentration range of 2.5 to 200 μM, with a limit of detection (LOD) of 0.74 μM and strong selectivity against interfering antioxidants. The sensor performance was validated using red and white wine samples from the local market, with results corroborated by HPLC, underscoring its potential for commercial and quality control applications.

## Results and Discussion

**Figure 1:**
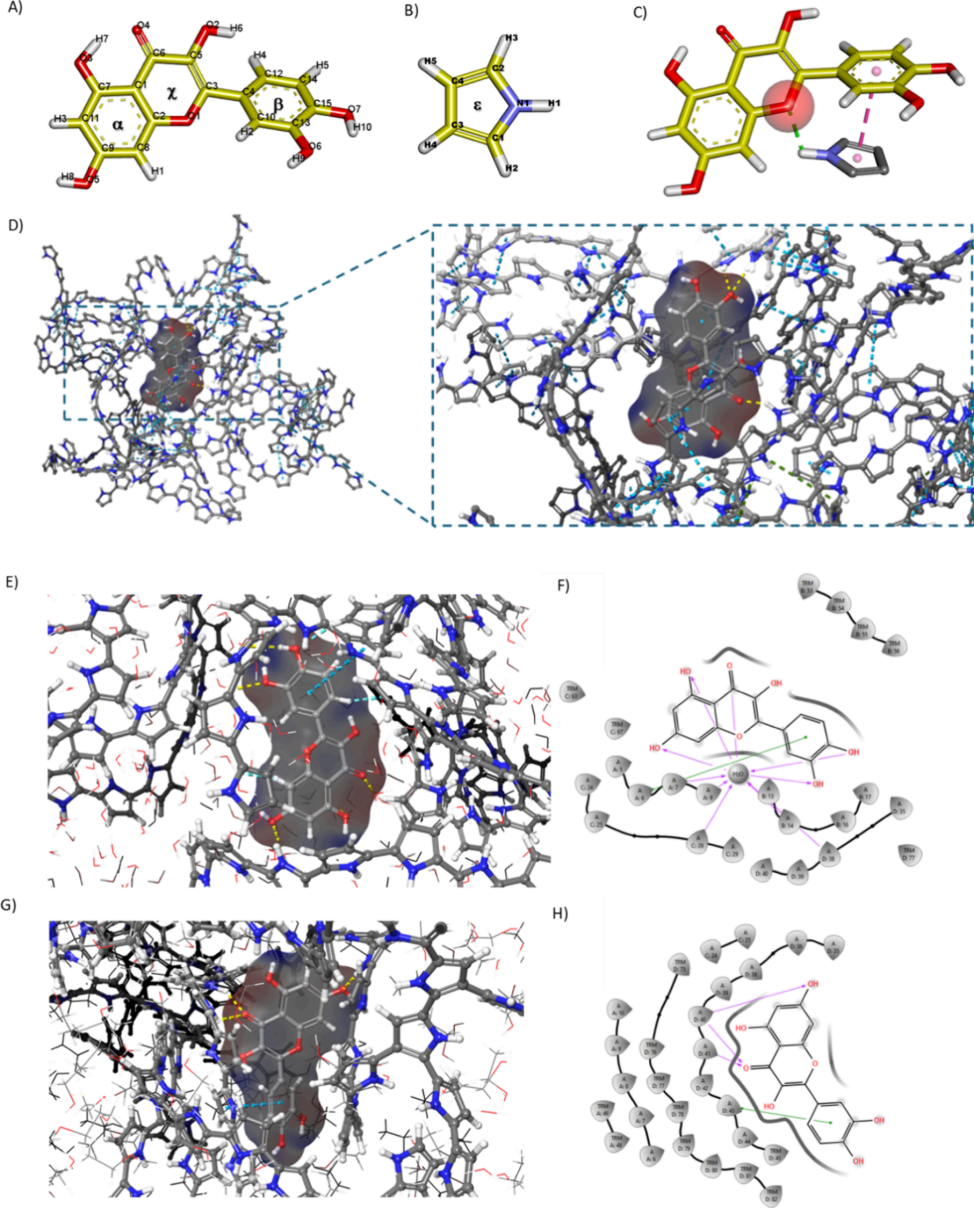
Optimized geometry of A) quercetin and B) pyrrole. The orange/gold coloration represents the carbon backbone of the molecules, while blue, red, and white indicate nitrogen, oxygen, and hydrogen, respectively. C) interactions between QU and Py by MM simulations. (D) Binding interactions of quercetin with an amorphous polypyrrole matrix highlighting π–π stacking (blue), hydrogen bonding (yellow) and π- cation interactions (green) that contribute to a stabilized binding configuration. (E-H) Molecular interactions of quercetin with the amorphous polypyrrole matrix in different solvents. (E) and (G) panel show interactions in a water-solvated system and a water–ethanol mixture, respectively. 3D representation of the docked pose of quercetin within the polypyrrole network, highlighting π–π stacking (blue), hydrogen bonding (yellow) and aromatic-H bond interactions (light blue). (F) and (H) 2D interaction diagrams of the highest-ranked docking pose, with purple dashed lines indicating hydrogen bonds and green lines representing π–π stacking.

Computational chemistry was initially employed to evaluate the interactions between the functional monomer pyrrole and QU, assessing binding feasibility and monomer-target affinity. MIP selective binding capacity depends on the number and nature of intermolecular interactions with the target.^39,40^ Functional groups within the monomer/polymer matrix play a critical role in determining binding strength and kinetics.^41^ To ensure accurate molecular interaction predictions, we performed Density Functional Theory (DFT) calculations to optimize the molecular structures of both QU and pyrrole (Figure 1A, B). DFT optimization reduces geometric distortions, enhancing the accuracy of molecular docking and binding energy estimations. Structurally, QU consists of two aromatic rings (α and β) bridged by an oxygen-containing heterocyclic ring (χ), while pyrrole is a nitrogen-containing heterocycle (ξ). Following DFT optimization, Molecular Mechanics (MM) calculations were performed using the AMBER force field to quantify monomer–template interactions, considering key noncovalent forces such as hydrophobic interactions, hydrogen bonds, and van der Waals forces. These forces collectively determine the overall binding energy (Δ*E*_*binding*_),^42^ calculated as
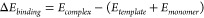
Where E_complex_, E_template_ and E_monomer_ represent the energy of binding complex, template, and monomer, respectively.

The favorable interaction energy (Δ*E*_*binding*_ = −1.75 kcal/mol) for the QU-pyrrole complex (Figure 1C) reflects a lower energy state compared to the sum of the individual monomer and template energies, suggesting a stable configuration. This decrease in binding energy is driven by hydrogen bonding between the heterocycle oxygen in QU and pyrrole nitrogen, along with π–π stacking interactions between the aromatic rings of QU and pyrrole, as shown in Figure S1A. The directional nature of hydrogen bonds contributes to the formation of binding moieties that mimic the target spatial configuration, thereby enhancing MIP selectivity.^43^ Although less directional, π–π stacking interactions are also beneficial for QU imprinting as they significantly enhance the stability of the binding complex, especially in environments where hydrogen bonds may be weakened, such as in aqueous solutions. Furthermore, π–π interactions enhance the stability of the prepolymerization complex, which is essential for efficient imprinting of the target molecule.

Further insight into the binding affinity between QU and pyrrole is provided by Mulliken atomic charge analysis and Molecular Electrostatic Potential (MeP) mapping.^44^ The distribution of the Mulliken charges and the orientation of the polar moment are shown schematically in Figure S1B. Highly negative Mulliken charge densities, particularly at QU carbonyl groups align well with the electropositive hydrogens of pyrrole reinforcing the strong potential for hydrogen bonding. MeP mapping further confirms these findings, highlighting several areas of high electrostatic potential (negative and positive regions) conducive to binding interactions (Figure S1B).

The binding interactions of QU with polypyrrole (PPy) were further explored at the polymer level (Figure 1D), offering insights into the intricate network of stabilizing forces within the amorphous PPy matrix. At this scale, the π–π stacking interactions between the aromatic rings of QU and the conjugated polypyrrole backbone emerged as a dominant stabilizing factor. These interactions are particularly crucial in maintaining the stability of the polymer-template complex, as they create an extensive network of noncovalent forces that promote target recognition and binding fidelity, even in competitive solvent environments. In addition to π–π stacking, hydrogen bonding interactions were also validated, primarily involving the hydroxyl and carbonyl groups of QU and the nitrogen atoms within the PPy chains. These interactions contribute to the structural integrity of the binding cavities, enhancing the selective and specific accommodation of the target molecule. The favorable binding environment provided by the PPy matrix was supported by molecular docking studies, where QU demonstrated a well-defined binding pose within the polymer framework. These simulations can decipher the spatial arrangement of the polypyrrole chains and optimal alignment of QU’s functional groups with the polymer’s binding sites, facilitating both hydrogen bonding and π–π stacking. This cooperative interaction network not only stabilizes the binding configuration but also enhances the imprinting efficiency by forming cavities that precisely match the target’s geometry.

To elucidate the impact of solvent environments on the binding of quercetin (QU) to polypyrrole based MIPs, molecular docking was conducted with solvated systems of amorphous polymers (Figure S2) - containing either pure water or a water–ethanol mixture (4:1 ratio). These simulations revealed favorable interactions and binding affinities in both the solvents, whereas the water–ethanol mixture demonstrated a more favorable binding environment, with a docking score of −7.32, compared with the water-solvated system (Figure 1E-H) where a docking score of −5.78 was recorded. Indeed, QU exhibited hydrogen bonding interactions with water, which potentially competes with its direct bonding to PPy chains, resulting in a lower binding score compared to water–ethanol system. Notably, ethanol allowed for better packing of QU within the polymer’s matrix and likely disrupted the structured water shell around QU, promoting closer contact with the polymer.

**Figure 2:**
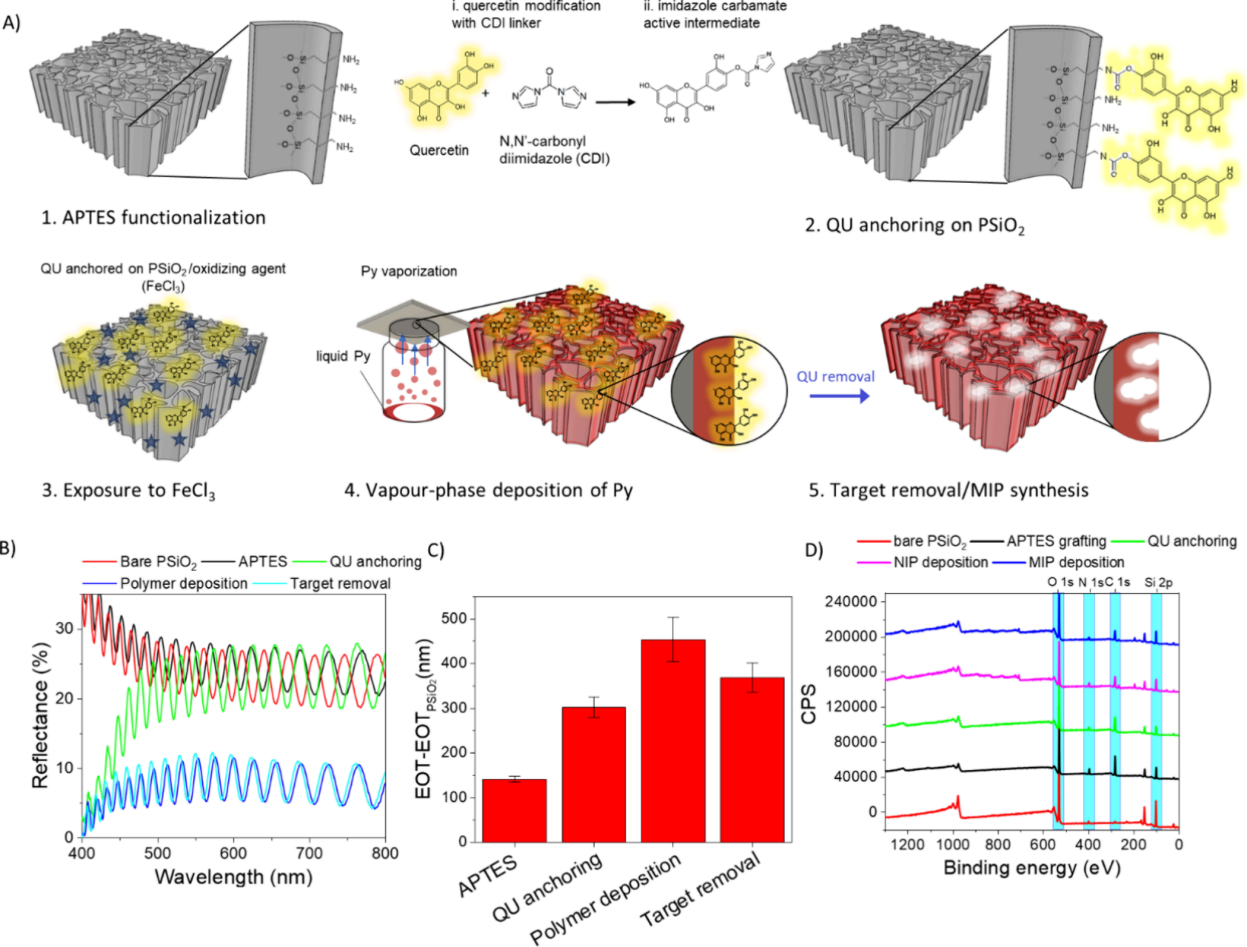
A) Sketch of the vapor-phase polymerization process, starting from PSiO_2_ scaffold functionalized with APTES (1), then exposed to an imidazole carbamate active intermediate for QU anchoring (2). The sample is then impregnated with oxidizing agent FeCl_3_ and then exposed to Py vapors to initiate the polymerization (3). Finally, PPy-coated PSiO_2_ (4) is subjected to a washing procedure to remove the target and then to obtain a MIP (5). B) Reflectance spectra recorded in air on a PSiO_2_ scaffold before and after each functionalization step up to the MIP synthesis. C) Effective optical thickness changes (EOT-EOT_PSiO2_) achieved for each functionalization step; the EOT value of bare PSiO_2_ (EOT_PsiO2_) scaffold is used as reference (*n* = 3 samples). All data are presented as mean (±s.d). D) Survey XPS scans recorded after each functionalization step.

**Figure 3:**
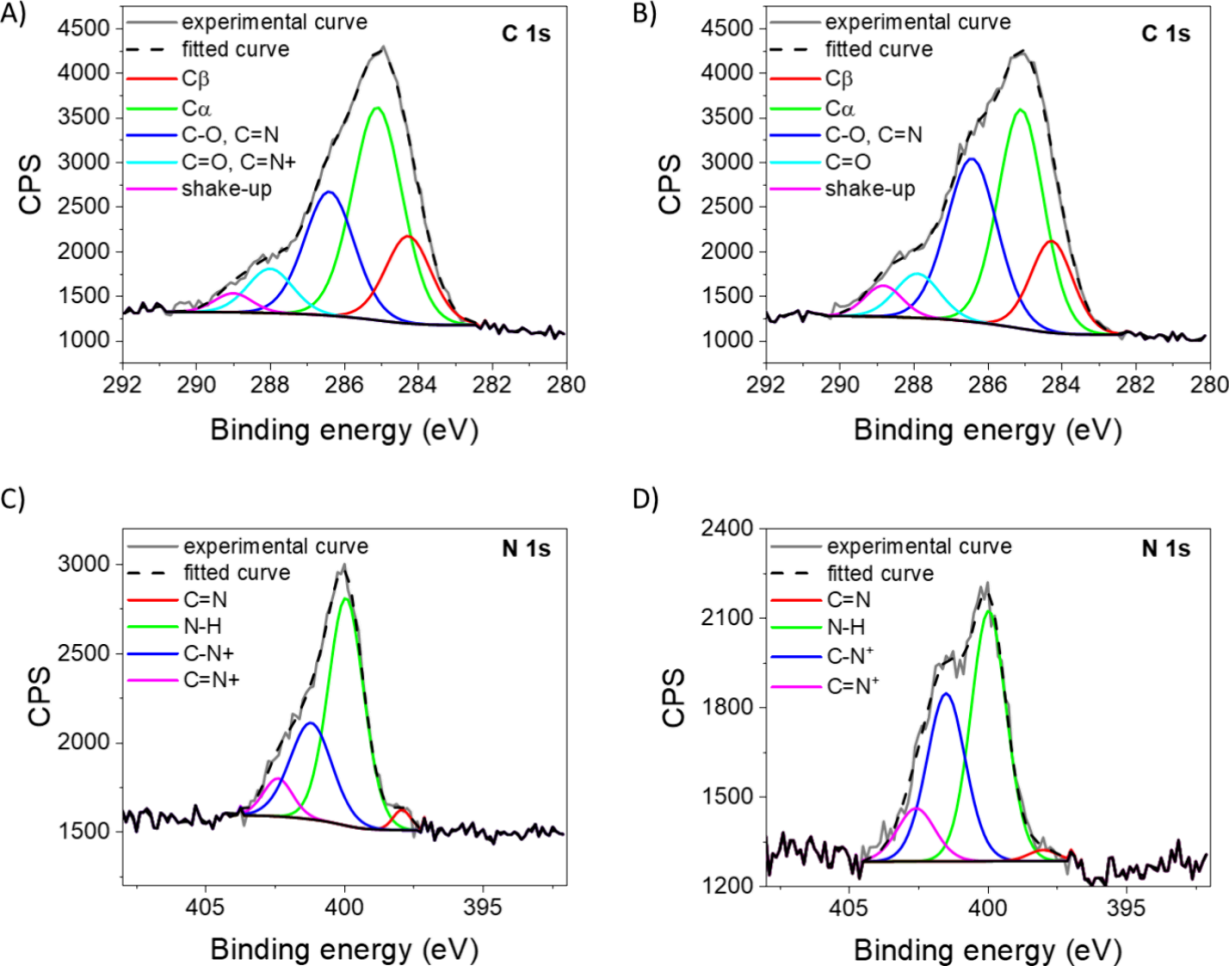
Detailed C 1s signals recorded for A) NIP- and B) MIP-deposited on PSiO_2_ scaffolds by vapor-phase deposition for 30 min. Detailed N 1s signals recorded for C) NIP- and D) MIP-deposited on PSiO_2_ scaffolds by vapor-phase deposition for 30 min. Spectra are fitted and charging corrected.

We then carried out vapor-phase deposition of polypyrrole (PPy) on nanostructured porous silica scaffolds (PSiO_2_) for the molecular imprinting of quercetin using a multistep process schematically reported in Figure 2A. Initially, the PSiO_2_ surface was functionalized via silanization with (3-aminopropyl)triethoxysilane (APTES) to anchor amino groups (Figure 2A-1), facilitating the subsequent attachment of QU. APTES molecules form stable covalent bonds with silica through silanol groups,^45^ providing robust surface modification essential for sensor stability and ensuring compatibility with molecular imprinting strategies.^29,46^ An original coupling chemistry protocol was used for anchoring QU to the silanized silica surface, consisting in the preliminary reaction between QU and 1,1′-carbonyldiimidazole (CDI) to form a stable imidazole carbamate active intermediate (Figure 2A-2). This intermediate acts as a linker, connecting hydroxyl (−OH) groups of QU with silane amino (−NH_2_) groups on the PSiO_2_ surface. This process ensures controlled molecular orientation and immobilization of QU through covalent bonding with amines, accompanied by the release of imidazole. Following QU anchoring, pyrrole polymerization on the PSiO_2_ scaffold was carried out via vapor-phase synthesis, upon exposure to the oxidizing agent FeCl_3_ in a chamber saturated with pyrrole vapors for various deposition times, namely, 30, 60, and 120 min (Figure 2A-3). After PPy deposition, the target molecule, i.e. QU, was removed through a simple washing procedure in TRIS buffer at pH 9, which induces degradation of QU leveraging its instability at pH levels above 7.5^47^ and promotes modification of the protonation state of the PPy film,^48^ facilitating complete target removal and subsequent formation of MIP binding sites. A nonimprinted polymer (NIP) was also prepared as a control material using the same procedure as the MIP, but without the target molecule. To ensure a proper comparison with the MIP, the PSiO_2_ scaffold was functionalized with APTES and CDI, omitting the QU anchoring step.

The functionalization of the PSiO_2_ was monitored using UV–vis reflectance spectroscopy, Raman and Fourier-transform infrared (FTIR) spectroscopy, and X-ray photoelectron spectroscopy (XPS) to confirm and characterize surface modifications.

The reflectance spectra of PSiO_2_ scaffolds recorded before and after functionalization steps are shown in Figure 2B, while Figure 2C illustrates the changes in effective optical thickness (EOT) as obtained from Fast Fourier Transform of the reflectance spectrum and quantified as EOT = 2*n*d, where *n* is the effective refractive index and *d* is the thickness of the porous layer.^29,49^ After APTES silanization, the reflectance spectrum red-shifted (Figure 2B) and an increase of EOT was recorded (Figure 2C). The QU anchoring via CDI produced a further red shift of the reflectance spectrum and an additional increase of EOT (about 150 nm), along with noticeable spectral modifications in the wavelength range 400–500 nm, due to QU absorption,^50^ further corroborating the successful QU anchoring to the PSiO_2_ surface. As a control, silanized PSiO_2_ was exposed to CDI alone resulting in a red-shift, which suggests CDI binding to APTES amino groups (Figure S3A). Vapor-phase deposition of PPy on the QU-functionalized PSiO_2_ scaffolds led to an additional red shift of the reflectance spectrum, accompanied by an increase in the EOT value, confirming the successful deposition of a thin PPy layer on the PSiO_2_ inner surface. The reduction in reflectance intensity is attributed to PPy absorption, further supporting the successful incorporation of the polymer layer. The thickness of the PPy film deposition increased with the deposition time, as indicated by the linear increase in the EOT values for times in the range 30 min to 2 h (Figure S3B-C). Washing of the PPy-coated samples to remove the QU from the polymer film caused a blue shift of the reflectance spectra and a corresponding decrease in the EOT value, regardless of the polymerization time (Figure S3C), which correlates with a reduction of the effective refractive index confirming the successful removal of QU from within the PPy film. As a control, the same washing procedure was performed on NIP samples, prepared under the same experimental conditions of MIP but avoiding QU anchoring on the PSiO_2_ surface prior polymer deposition. Figure S4 compares the functionalization steps of NIP- and MIP-sensors prepared performing a 30 min vapor-phase polymer deposition. The differences observed between MIP and NIP after the washing step strengthen the conclusion that the EOT reduction in the MIP is specifically due to the removal of the target molecule from the polymer matrix and not to any polymer instability effects (Figure S4).

XPS analyses were performed to monitor each functionalization step leading to MIP deposition within the PSiO_2_ layer. Wide spectra (Figure 2D) revealed significant changes in the C 1s, N 1s, O 1s, and S 2p signals after each functionalization step. Following APTES silanization, an increase in C 1s and N 1s signals was evident. Detailed C 1s analysis (Figure S5A) showed a peak at 284.8 eV, corresponding to the hydrocarbon chain of APTES, and a higher binding energy peak at 286.4 eV, likely representing C–N and residual C–O ethoxy groups. The N 1s spectrum (Figure S5B) included a peak at ∼399 eV (−NH_2_ groups) and another at ∼401 eV, attributed to quaternary nitrogen from proton transfer between surface silanols and amino groups.^51^

The successful anchoring of quercetin via the CDI linker was confirmed by XPS, with clear changes observed in the high-definition C 1s and N 1s spectra (Figure S4C-D). Fitting of the C 1s spectrum (Figure S5C) reveals the increase of component at 286.2 eV, due to numerous C–O groups of QU, and the appearance of two additional components at about 288.0 and 288.5 eV respectively ascribed to C = O of QU and to carbamate moieties originated from QU anchoring. The presence of carbamate groups indicative of QU binding is further evidenced by N 1s detailed spectrum (Figure S5D) exhibiting a pronounced peak at about 402 eV, along with a minor peak at 399.6 eV, corresponding to amine groups. Control experiments exposing APTES-functionalized PSiO_2_ to CDI only confirmed these assignments, showing N 1s and C 1s profiles evidently lacking components attributed to QU (Figure S4E-F).

XPS analysis of MIP- and NIP-functionalized PSiO_2_ scaffolds after 30 min of vapor-phase polymerization was then carried out. The comparison of high-resolution C 1s spectrum of NIP (Figure 3A) and MIP (Figure 3B) revealed the same components with an evident increase of the peak at 286.3 eV in MIP, possibly reflecting the incorporation of quercetin into the polymer matrix, in agreement with results on QU-anchored PSiO_2_ (Figure S5C). Interestingly, the analysis of N 1s spectra in NIP (Figure 3C) and MIP (Figure 3D) leads to the same conclusions, evidencing in both cases peaks ascribed to polypyrrole functionalities,^29^ with the component at about 402 eV more pronounced in MIP, suggesting the presence of QU in the polymer.

C/Si and N/Si atomic ratios were evaluated for each functionalization step (Figure S6). Notably, the C/Si ratio increase from 1.88 (after silanization with APTES) to 2.65 after QU anchoring (Figure S6A), reflecting the carbon-rich framework of quercetin, along with a remarkable increase of N/Si ratio after MIP and NIP deposition due to pyrrolic nitrogen on the PSiO_2_ scaffolds.

**Figure 4:**
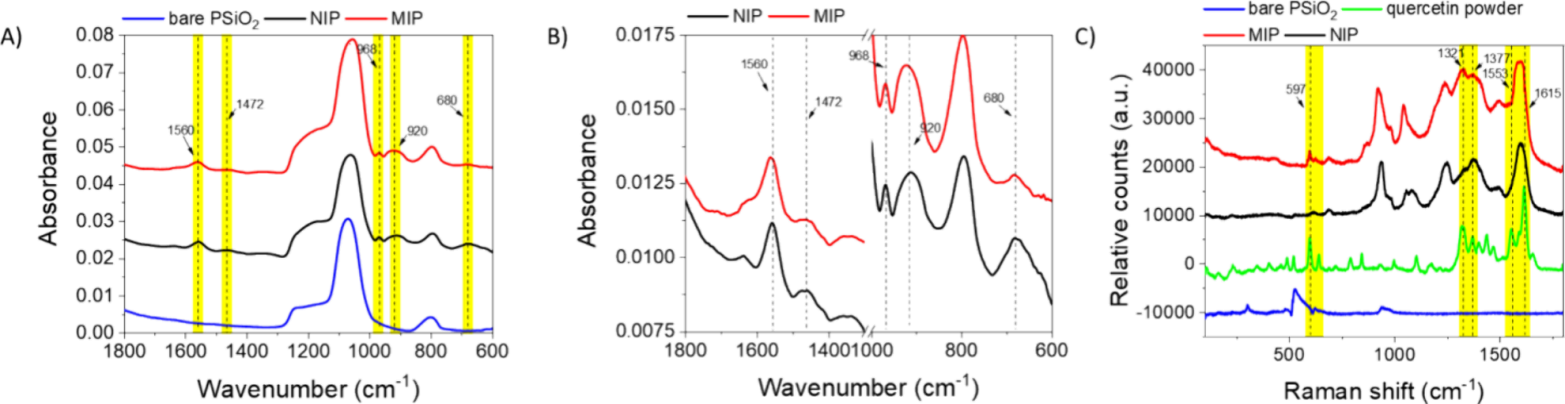
A) FT-IR spectra of bare PSiO_2_ (blue line), NIP-coated (black line) and MIP-coated (red line) PSiO_2_ scaffolds recorded from 600 to 1800 cm^–1^. The NIP and MIP traces are offset along the *y*-axis by 0.02 and 0.04 absorbance units, respectively; B) Scale adjustment for the FT-IR spectra of NIP-coated (black line) and MIP-coated (red line) PSiO_2_ scaffolds from 600 to 1800 cm^–1^. The MIP trace is offset along the *y*-axis by 0.002; C) Raman spectra of bare PSiO_2_ (blue line), NIP-coated (black line) and MIP-coated (red line) and quercetin powder, recorded from 150 to 1800 cm^–1^.

Together with XPS analysis, NIP- and MIP-coated PSiO_2_ scaffolds were further characterized by FT-IR and Raman spectroscopy (Figure 4).

The FT-IR spectrum of the unmodified PSiO_2_ scaffold (Figure 4 A) displays a prominent peak at approximately 1050 cm^–1^, with an extended shoulder up to around 1300 cm^–1^, attributed to the stretching vibrations involving the in-phase and out-of-phase motions of oxygen atoms in the Si–O–Si bond.^52^ Additionally, a broad peak at around 800 cm^–1^, indicative of Si–O bending vibrations, is observed.

The vapor-phase deposition of MIP and NIP on PSiO_2_ scaffolds led to detectable modifications in the FT-IR spectrum (Figure 4 A, B). In Figure 4B the magnification of selected ranges of these spectra clearly shows the characteristic peaks for PPy formed by the oxidative polymerization of pyrrole. The peak at 680 cm^–1^ corresponds to the C–C out-of-plane ring deformation vibration, while the peak at 920 cm^–1^ can be attributed to C–H wagging or out-of-plane ring deformation. Peaks at 1560 and 1472 cm^–1^ represent the C = C and C–N stretching vibrations of pyrrole, in agreement with the literature.^53^ Nevertheless, the expected PPy peaks within the 1000–1300 cm^–1^ region are overshadowed by the broader SiO_2_ signal and are not discernible. No significant differences were observed between the FT-IR spectra of MIP and NIP.

The Raman spectrum of the bare PSiO_2_ scaffold shows three major peaks, with a prominent peak at ∼511 cm^–1^ corresponding to Si–Si stretching,^54^ a smaller vibrational peak at 230 cm^–1^ related to SiO_2_ tetrahedral scissoring, and a broad peak around 930 cm^–1^ attributed to multiphonon scattering in the Si substrate, as reported in the literature.^54^

The Raman spectrum of the NIP-coated PSiO_2_ scaffold reveals distinct PPy peaks^55^ (Figure 4C). The peak at ∼937 cm^–1^, with a shoulder extending to 978 cm^–1^, is attributed to bipolaron and polaron ring deformation vibrations, confirming pyrrole’s chemical oxidation during polymerization.^56^ The C–H vibrations are at 1059 and 1084 cm^–1^ and at 1248 cm^–1^. The antisymmetric C–N stretching, N–H bending, C–H bending, and C–C ring stretching vibrations are the components of the broad peak around 1375 cm^–1^. The 1492 and 1603 cm^–1^ peaks are relevant to C = N and C = C stretching, respectively.^57^

A comparable Raman profile was observed for the MIP-coated PSiO_2_ scaffold, although some notable differences indicate the presence of quercetin within the MIP matrix. For instance, a small vibrational peak at 597 cm^–1^, absent in the NIP, is associated with the out-of-plane bending of the quercetin phenyl ring.^58^ Additionally, quercetin peaks at 1321 and 1377 cm^–1^, related to O–H bending and C–C ring stretching,^59^ were detected. The other spectral region where quercetin entrapment in the polymer matrix is evident is between 1550 and 1650 cm^–1^ range where the C = C stretching modes of the phenol derivative at 1553 and 1615 cm^–1^ are evident.

Overall, the FT-IR analysis did not reveal significant differences between the MIP and NIP as, contrarily to Raman measurements, may be affected by PPy absorbance possibly masking the peaks related to the target.

**Figure 5:**
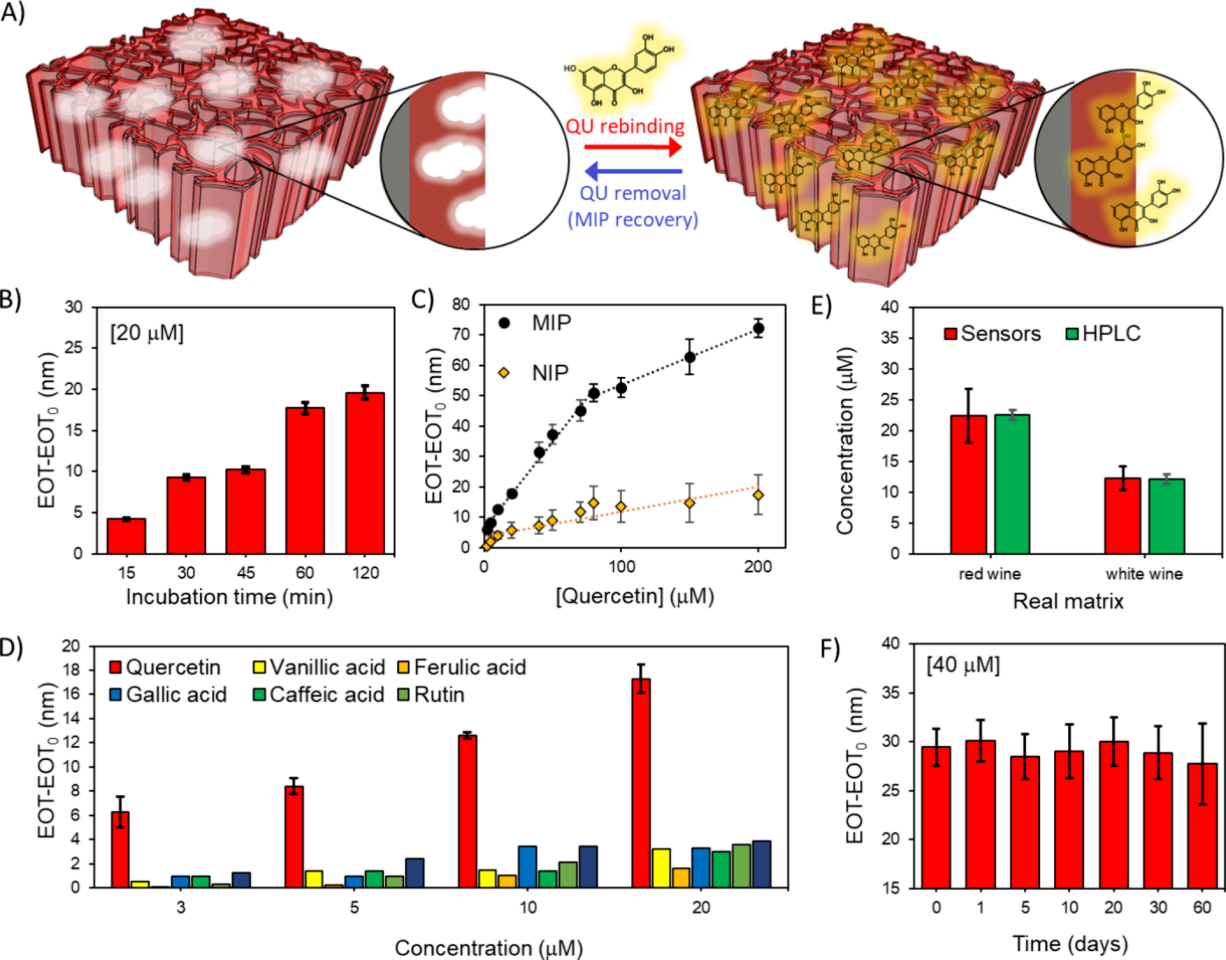
A) PPy-based MIP sensing performance in QU optical detection. a) Schematic of QU target interaction with PPy-based MIP on PSiO_2_ scaffold. B) Sensor response (EOT-EOT_0_) recorded after the incubation of MIP-functionalized PSiO_2_ scaffolds for different contact times, namely 15, 30, 45, 60, and 120 min; EOT_0_ is the signal recorded for the blank solution and used as reference. QU concentration: 20 μM. MIP polymerization time: 30 min (*n* = 3 samples) C) Calibration curves (EOT-EOT_0_ vs QU concentration) recorded on MIP and not imprinted polymer (NIP) sensors in the range 2.5 to 200 μM. EOT_0_ is measured in buffer solution and used as reference (*n* = 3 samples). D) Selectivity results comparing the MIP sensor response (calculated as EOT-EOT_0_) to QU and to interfering molecules at different concentrations (*n* = 3 samples). E) MIP sensor response (calculated as EOT-EOT_0_) in real samples of red and white wines compared with HPLC results (*n* = 3 samples). F) MIP sensor response (calculated as EOT-EOT_0_) versus time measured over 60 days at QU concentration of 40 μM. (*n* = 3 samples). All data are presented as mean (±s.d).

We then assessed the MIP-coated PSiO_2_ scaffolds for their ability to detect QU (Figure 5A). MIPs prepared for different polymerization times (30 min, 1 and 2 h) were tested for optical detection of QU at concentrations in the range 2.5–20 μM in water (Figure S7A). The highest sensitivity was observed with MIPs polymerized for 30 min, which were chosen for subsequent experiments. To further evaluate sensor performance, QU detection tests were carried out at the same concentrations in water/ethanol (4:1) mixture, a matrix in composition closer to wine. No significant differences were observed between water and the ethanol mixture (Figure S7B), evidencing the suitability of the sensor to work in alcoholic solutions. The slightly higher signals in the ethanol mixture may be attributed to the to the interaction mechanisms between the PPy-based polymer and QU, involving hydrogen bonding and π–π stacking interactions, which computational simulations suggest are enhanced in less polar environments. This consistency highlights the robustness of the developed system across varying media.

Kinetic binding studies were also conducted on the MIP-coated PSiO_2_ scaffolds at 20 μM of QU, monitoring EOT changes over incubation times from 15 to 120 min. As shown in Figure 5B, the binding signal increased up to 60 min, then reached a plateau likely due to a saturation of MIP binding sites. Based on these results, a 60 min incubation time was selected for subsequent binding studies to ensure sufficient interaction between the MIP and QU.

MIP- and NIP-coated (30 min polymerized) PSiO_2_ scaffolds were extensively tested for QU detection across concentrations ranging from 2.5 to 200 μM. Reflectance spectra were recorded after a 60 min incubation and the change in the EOT value, namely, EOT-EOT_0_, was used as the analytical signal, with EOT_0_ reference value measured in buffer solution (Figure 5C). The concentration range was selected based on the typical variation of quercetin content in beverages, particularly red wines.^60^ The MIP sensor exhibits a dual linear response: a high-sensitivity range (0.57 nmμM^–1^, R^2^=0.992) between 2.5 to 80 μM and a low-sensitivity range (0.18 nmμM^–1^, R^2^=0.993) between 80 and 200 μM. This behavior suggests the existence of two populations of binding sites with different affinities within the MIP matrix. To explore this aspect further, the calibration curve was fitted to the Freundlich model (Figure S8), a commonly used approach to describe a continuous range of binding sites with varying affinities in MIPs.^61^ The Freundlich isotherm describes the sensor output EOT-EOT_0_ as a power function of the quercetin concentration C in solution, as follows:
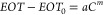
1

The parameter *a* is the Freundlich coefficient related to binding affinity, and *m* is the heterogeneity index. The value of *m* ranges between 0 and 1, where *m* < 1 indicates a heterogeneous material and m = 1 signifies a homogeneous sorption material. The estimated R^2^ value of 0.982 indicates a good fit of the model to the experimental data, and the *m* value of 0.53 suggests a significant degree of heterogeneity in the binding sites, consistent with the presence of two distinct linear response ranges in the sensor calibration curve. The MIP-based sensor demonstrated significantly higher responses than the NIP-based sensor across the whole concentration range. The imprinting factor (IF), calculated as the ratio between the sensitivity of the MIP to that of the NIP in the range 2.5–80 μM, was 3.6, exceeding previously reported values for MIPs designed for QU detection.^19,20^ The sensor limit of detection (LOD) and limit of quantification (LOQ) evaluated as 3.3σ/S and 10σ/S (where σ is the standard deviation and S is the slope of the calibration curve) were 0.7 and 2.3 μM, respectively, making the sensor suitable for detecting and quantifying QU concentrations in wine samples.^62^ The sensor reproducibility, evaluated by comparing the sensitivity of three different MIP-based sensors, showed a satisfactory variability with a relative standard deviation (RSD%) of 9.5%.

Beverages as wine are rich in various flavonoid and flavonoid-like compounds, making selectivity a critical parameter for reliable sensing performance in real sample analysis.^63^ MIP selectivity has been tested against other bioflavonoids and antioxidant molecules, including rutin, luteolin, vanillic acid, ferulic acid, gallic acid, and caffeic acid, which are structural analogs of quercetin commonly found in wine. The response to QU (Figure 5D) is at least five times higher than the other compounds, even at the lowest concentration. Remarkably, this trend is particularly significant for luteolin and rutin, which are known to be challenging interferents in quercetin detection.^64^

We further assessed the sensor response in real samples of “Negroamaro Salento IGT” and “Chardonnay Salento IGT”, respectively a red and white wine from the Salento region, without prior sample preparation (Figure 5E). The use of PSiO_2_ scaffolds is highly beneficial in real sample analysis as the nanoporous layer acts as a filter for particulate and suspended matter in the matrix, which cannot reach the MIP binding sites. Wine often contains suspended microparticles resulting from the vinification process or aging, such as tartrate microcrystals^9^ or fermentation-associated microbiota,^65^ which can interfere with the target analyte detection. The sensor output in wine samples was validated by comparison with high-performance liquid chromatography (HPLC) analysis of the red and white wines (Figure S9), using an established literature method for QU detection in wine.^66^ The strong correlation between the sensor response and HPLC results confirmed the sensor reliability and robustness for QU detection in complex wine matrices (Figure 5E).

Eventually, we investigated the sensor reusability and stability over multiple use and time. Reusability was tested by performing three consecutive measurements at the lowest concentration (2.5 μM), with a simple 5 min wash in TRIS buffer at pH 9 between each measurement to regenerate the MIP. The sensor exhibited excellent repeatability, with an RSD of only 2.6%. To assess stability, the sensor response was monitored over 60 days, highlighting high sensor response stability with an RSD of approximately 3%. The results demonstrated high stability, with an RSD of approximately 3%, confirming the sensor robustness and suitability for long-term applications (Figure 5F).

The sensor performance was compared with other MIP-based sensors for quercetin reported in the literature (Table 1).^19−22,24,67,68^

**Table 1:** Comparison of the Performance in QU Detection between the Sensor Developed in this Work and Other State-of-the-Art MIP^a^

**MIP synthesis approach**	**Detection Method**	**Concentration range tested (μM)**	**LOD (μM)**	**Imprinting factor (IF)**	**Real samples analysis**	**Stability over time (days)**	**References**
free radical polymerization	optical (fluorescence)	10–50	0.03	1.8.	*Ginkgo biloba* extracts(after sample pretreatment)	N.D.	(19)
thermally initiated radical polymerization	optical (fluorescence)	0.06–3	0.005	2.3	Urine and onion skin (after samples pretreatment)	N.D.	(20)
Precipitation polymerization	optical (chemiluminescence)	1.5–160	1	N.D	Quercetin capsules	N.D	(67)
Sol–gel polymerization	optical (fluorescence)	15–70	1.7	4.2	Green tea, cumin and thyme	N.D.	(21)
Electro-polymerization	electrochemical (DET)	0.005–10	0.00015	N.D.	Quercetin capsules (after sample pretreatment)	30	(22)
Thermal polymerization	electrochemical (DET)	0.001–400	0.00013	N.D.	Food samples, onion, oregano, spinach	30	(68)
Electro-polymerization	electrochemical (DET)	0.5–15	0.05	N.D.	Apple juice (after sample pretreatment)	15	(24)
Vapor-phase polymerization	optical (UV–vis spectroscopy)	2.5–200	0.74	*∼* 4	Red and white wines	60	this work

a N.D.: not declared; DET: direct electrochemical detection monitoring electro-oxidation of quercetin.

Key advantages of the optical MIP-based sensor proposed in this study include the wide concentration range, and the ability to operate in real matrices without pretreatment, which enable its possible application in different real samples. The high sensor reliability, evidenced by data validation through HPLC analysis, and the excellent long-term stability are additional beneficial features of the sensor. Also, the easy sensor assembly, requiring 30 min polymerization, and the possibility of extending the synthetic approach to other target and other transducers, highlight the sensor potential for practical applications in quality control and monitoring of different compounds in wine and other complex food matrices.

## Conclusions

In this study, we developed a robust, sensitive, and selective MIP-based optical sensor for the detection of quercetin (QU) in red and white wines. By combining the selective recognition properties of molecularly imprinted polymers (MIPs) with the optical advantages of nanostructured porous silica (PSiO_2_) scaffolds, the sensor achieved reliable detection of quercetin in both simple aqueous matrices and complex wine samples without requiring prior sample preparation. The integration of a vapor-phase polymerization method ensured a homogeneous MIP layer with precise thickness control, enhancing sensor performance.

The sensor demonstrated dual linear response ranges, high sensitivity, and a low detection limit (0.7 μM), making it suitable for detecting the varying concentrations of quercetin commonly found in wine. Selectivity tests confirmed its ability to distinguish quercetin from structurally similar flavonoids and antioxidants, with a response at least five times higher for quercetin than for potential interferents such as luteolin and rutin. Validation against high-performance liquid chromatography (HPLC) showed a strong correlation, reinforcing the sensor’s reliability for real-sample analysis.

The sensor also exhibited excellent reusability and long-term stability, with minimal variability in signal over consecutive uses (RSD = 2.6%) and across 60 days of storage (RSD = 3%). These findings highlight the sensor potential for practical applications in quality control and monitoring of bioactive compounds in wine and other complex food matrices.

Overall, this work demonstrates the feasibility of MIP-based optical sensors as a cost-effective and efficient alternative to conventional analytical methods, paving the way for real-time monitoring of key antioxidants and bioactive compounds in the food and beverage industry.

## Materials and Methods

### Reagents

All chemicals were of analytical grade and were used as received. Ultrapure water (conductivity <0.1 μS/cm) obtained from Labostar Pro UV 2 (Evoqua, Günzburg, Germany) was used.

The chemical reagents used included (3-aminopropyl)triethoxysilane (APTES), 99%, quercetin dihydrate powder (QU), 99%, 1,1′-carbonyldiimidazole (CDI), 99%, rutin (RU) dihydrate powder, 99%, gallic acid (GA), 99%, dimethyl sulfoxide anhydrous (DMSO), ≥ 99.9% and vanillic acid (VA), 99%, obtained from Sigma-Aldrich (St. Louis, MO, USA). Iron chloride, sodium hydroxide, monosodium phosphate (MSP), NaH_2_PO_4_, and disodium phosphate (DSP), Na_2_HPO_4_, were provided from Honeywell Fluka (College Park, GA, USA); Diethyl ether (Et_2_O, > 99%), methanol, ethanol, isopropanol of analytical grade were purchased from Carlo Erba (Milan, Italy).

Silicon boron-doped wafers (p++ type) with resistivity of 0.8–1.2 mΩ × cm, orientation <100>, were purchased from Siltronix Silicon Technologies (France). Laboratory-grade nitrogen gas (purity ≥99.999%) was used as inert gas to dry the samples after each functionalization step.

All solutions (except APTES and CDI) were prepared in ultrapure water. Phosphate buffer saline (PBS) solutions (50 mM, pH 7.4), were prepared by dissolution of the commercial MSP and DSP in appropriate proportions, adding NaOH 5 M to adjust the final pH.

CDI solutions (5 mg mL^–1^) were prepared in acetone. Stock solution of QU (30 mg mL^–1^) was prepared in DMSO and was diluted in water or EtOH/water (1:4, v/v) for obtaining QU standard solution at different concentrations (from 2.5 to 200 μM) for rebinding experiments. Solutions of rutin, luteolin, gallic acid and vanillic acid, were freshly prepared in a similar manner, before their use.

### Computational Predictions of the Prepolymerization Complex

The two-dimensional structures of quercetin (QU) and pyrrole (Py) were obtained from the PubCHEM online database (https://pubchem.ncbi.nlm.nih.gov) and subsequently converted into three-dimensional structures using Chem3D software (version 20.1). Geometry optimization for these 3D structures was carried out through density functional theory (DFT) calculations implemented in Gaussian16 (Gaussview 6, Gaussian, Inc., Wallingford, CT USA).^69^ The optimization utilized the B3LYP functional with the 6-311++G(d,p) basis set, ensuring energy minimization at the lowest possible energy state. The B3LYP functional—Becke’s three-parameter hybrid DFT with Lee–Yang–Parr correlation—was chosen for its capability to accurately predict thermochemical properties and describe electron exchange-correlation at a theoretical level.

Molecular docking simulations between QU and Py were performed using AutoDock Vina (from AutoDockTools 1.5) to evaluate their interactions. The results, including the position and type of bonds formed as well as the binding affinity of the template/monomer prepolymerization complex, were analyzed with AutoDockTools 1.5. Visual representations and further analysis of the docking outcomes were generated using Chimera software (UCSF Chimera, University of California). The Mulliken charge distribution for QU and Py molecules was calculated using B3LYP/6-311++G(d,p) method and basis sets level calculations.

To construct the amorphous polypyrrole (PPy) polymer systems, Schrödinger’s Materials Science Suite (Suite 2025, Schrödinger, Inc.) was employed. Initially, pyrrole monomers were sketched in the polymer builder module and polymerized into amorphous structures using the amorphous cell builder module. The resultant polymer matrix was equilibrated and optimized to develop a model system of the PPy structure.

Short MD simulations (10 ns) were performed to further refine the polymer model and allow relaxation of the polymer chains, ensuring proper packing and structural stability of the amorphous PPy matrix. Next, the solvation builder module was utilized to introduce solvent molecules into the system. Two solvation environments were prepared: (i) pure water, containing 2000 water molecules, and (ii) a water–ethanol mixture in a 4:1 ratio, consisting of 1600 water molecules and 400 ethanol molecules. To facilitate docking studies, receptor grids were generated over the PPy chains using the receptor grid generation tool in Schrödinger’s Glide module. The grids were strategically placed to cover key regions of the polymer matrix. Finally, QU was docked onto the receptor grids using the Ligand Docking protocol. The docking simulations were conducted to evaluate the binding interactions between QU and the PPy matrix in a solvated system, providing insights into key stabilizing forces, including hydrogen bonding and π–π stacking, in different solvent environments. The optimal binding configurations and affinities of QU within the PPy systems were scored using Glide empirical scoring function, GScore (kJ/mol), that approximates the ligand-binding free energy and higher negative Gscore values indicate favorable binding.

### Porous Silicon (PSi) Substrate Preparation and Oxidation

PSi samples were prepared by a method already known in the literature.^70^ An electrochemical etching of silicon wafer (15 × 15 mm) was performed using a solution of hydrofluoric acid (48%) and ethanol (3:1, v/v). A two-electrodes Teflon cell with a platinum wire cathode and an aluminum flat anode was employed to electrochemically etch silicon samples over a circular area of 0.567 cm^2^ by using a Keithley 2602A SourceMeter, setting current density and measuring the voltage. A first PSi sacrificial layer was etched at 600 mA cm^–2^ for 10 s and dissolved by alkaline dissolution with a solution of NaOH(1 M) and EtOH (9:1, v/v). The silicon samples were rinsed with ultrapure water, ethanol and then dried under a gentle nitrogen flow. The PSi sensing layer (i.e., the PSi interferometer) was then etched at 600 mA cm^–2^ for 25 s on the so-processed silicon samples, rinsed with isopropanol and diethyl ether, and gently dried under a nitrogen flow to achieve a crack-free PSi layer. Thermal oxidation of the PSi interferometer was performed in muffle (Nabertherm, Lilienthal, Germany) at 1000 °C for 10 min (ramp-up/ramp-down 15 °C min^–1^) to obtain PSiO_2_ samples.

### Characterization of PSiO_2_ Scaffolds and FFT Reflectance Spectroscopy

Reflectance spectra of the PSiO_2_ interferometers were acquired in air in the wavelength range [400–1000 nm] using an optical setup consisting of a UV–VIS spectrometer (SM242 SP) provided by Spectral products, a bifurcated fiber-optic probe (QR200–7-VIS-BX) and a lamp source (HL-2000) provided by Ocean Optics (USA). Light from the halogen lamp source is fed orthogonally onto the PSiO_2_ surface and the light reflected from the PSiO_2_ layer is collected into a UV–VIS spectrometer by the fiber-optic probe. Acquisition parameters for reflection spectra were: integration time 50 ms, average scan number 15, boxcar width 5, with the spectrometer working in photon counts mode. Porosity of as-prepared PSiO_2_ scaffolds was estimated by best-fitting of the reflectance spectra of PSi layers acquired before oxidation.^70^ PSiO_2_ prepared from p-type silicon wafer exhibits well-defined Fabry–Perot fringes in the reflectivity spectrum whose position is governed by the relationship:
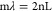
where m is the spectral order of the optical fringe, λ the wavelength at which each interference maximum appears, n the refractive index of the film, and L its thickness.

FFT of the reflectance spectra of PSiO_2_ scaffolds was performed to calculate the EOT values, namely, 2 nL, where n = effective refractive index and L = thickness of the PSi layer, using a homemade software (MatLab, R2024a, 24.2, MathWorks, USA). The wavelength axis of the reflectance spectrum was first inverted (*x* axis changed from wavelength to 1/wavelength) to obtain a wavenumber axis. A cubic-spline interpolation of reflectance data was then carried out to obtain a data set (reflection, wavenumber) spaced evenly (sample-to-sample distance 8.57 × 10^–7^ nm^–1^). A Hanning window was applied to the reflectance spectrum, which was zero padded to 224. Eventually, application of the FFT algorithm to the zero-padded reflectance spectrum yielded the Fourier transform amplitude and phase (*y* axis in the Fourier transform domain) as a function of 1/wavenumber (*x* axis in the Fourier transform domain), with spatial resolution of about 0.07 nm. The EOT value is obtained as the value of the 1/wavenumber axis (*x* axis) in the Fourier transform domain for which the main peak in the Fourier transform amplitude (*y* axis) occurs.

### QU Anchoring on PSiO_2_ Scaffolds

The target immobilization on PSiO_2_ scaffolds was based on the covalent anchoring of quercetin and involves a preliminary functionalization of silicon surface with a suitable linker. PSiO_2_ scaffolds were first cleaned by a treatment with piranha solution (H_2_SO_4_:H_2_O_2_, 3:1, v/v) for 10 min at 40 °C. Later, the samples were immersed in a solution of APTES prepared in toluene (2%, v/v) for 30 min at 55 °C, then washed with MeOH for 5 min and rinsed with water and EtOH. Next, the samples were immersed in a solution containing CDI (5 mgml^–1^) and quercetin (1 mgml^–1^) prepared in acetone, for an overnight step, producing target anchoring.

### PSiO_2_ Functionalization with Molecularly Imprinted Polymer Films

Vapor-phase polymerization of the MIP for QU was carried out optimizing a protocol reported in literature.^29^ The PSiO_2_ samples were immersed in a FeCl_3_-ethanol solution (0.5 wt %/v) for 20 min. After taking the samples out from the solution and gently drying them with a N_2_ flow, the PSiO_2_ samples were placed on the top of a 4-ml vial containing pyrrole vapors for 1 h at room temperature and atmospheric pressure, allowing the polymerization within the porous layer. Preliminary to the polymerization, 20 μL of pure Py were placed in the vial, allowing its vaporization at ambient conditions. After the polymerization, the samples were repeatedly rinsed with water (5 min) to remove unreacted monomer, then with ethanol, and dried under a N_2_ flow.

Subsequent removal of QU molecules from the polymer matrix by washing (1 h, stirring) with TRIS buffer at pH 9 prepared in water, produce the imprinted cavities and then, the MIP.

As reference, not-imprinted polymers (NIPs) were synthesized as previously described but using a polymerization solution without target molecules. Likewise with MIPs, NIPs were subjected to washing procedures.

### Binding Kinetics and Rebinding Tests

The effect of contact time (or incubation time) between MIP and target on the sensor response was evaluated in a time range of 15 to 120 min. Freshly prepared QU (50 μM) standard solutions prepared in water/EtOH were placed (20 μL) on PSiO_2_ scaffold functionalized with the quercetin imprinted polymer for different incubation times. After incubation, the sensors were washed with water (2 min, under stirring), rinsed with ethanol, and then dried under a gentle nitrogen flow. The reflectance spectra were recorded before and after the exposure of the sensor with the quercetin solutions. Before a new experiment, MIPs were restored by a washing procedure (10 min, under stirring) in TRIS buffer at pH 9.

During the rebinding tests, MIP-functionalized PSiO_2_ samples were exposed to increasing concentrations of target standard solutions (20 μL) prepared in water or water/EtOH (4:1, v/v) mixture (2.5 to 200 μM). The reflectance spectra were recorded before and after each QU concentration tested. All experiments were carried out in triplicate (n = 3).

### Selectivity, Repeatability, and Stability Tests

MIP selectivity was evaluated by testing MIP-coated PSiO_2_ sensors with solutions containing different interfering molecules such as vanillic acid, ferulic acid, gallic acid, caffeic acid, rutin and luteolin (2.5–20 μM). Fresh solutions were prepared immediately before the experiments.

Repeatability was assessed by testing the MIP-PSiO_2_ sensor for QU detection in three consecutive experiments with the same sensor. Again, to regenerate the MIP is sufficient a treatment with TRIS buffer at pH 9 for 10 min.

The time stability of the MIP-based sensor for QU detection was evaluated by monitoring the sensor response at 50 μM for different time intervals up to 60 days. The sensor was stored in the air without any particular care and washed for 10 min, before its use.

### X-ray Photoelectron Spectroscopy (XPS) Characterization

XPS measurements were conducted using an AXIS ULTRA DLD photoelectron spectrometer (Kratos Analytical, Manchester, UK) equipped with a monochromatic AlKα source (1486.6 eV) operating at 150 W (10 kV, 15 mA). The base pressure in the analysis chamber was maintained at 5.3 × 10^–9^ Torr. Survey spectra were recorded with a pass energy of 160 eV and a step size of 1 eV, while high-resolution spectra were acquired with a pass energy of 20 eV and a step size of 0.1 eV. The analysis area measured approximately 700 μm × 300 μm. A charge neutralization system was employed during data acquisition to mitigate surface charging. Spectral data were processed using CasaXPS software (version 2.3.16), with the binding energy (BE) scale calibrated to the Au 4f7/2 peak at 84.0 eV. High-resolution spectra were fitted with a Shirley background and a GL(30) line shape (70% Gaussian, 30% Lorentzian). Quantitative analysis was performed using the relative sensitivity factors provided in the CasaXPS library for signal areas. Surface charging was corrected by referencing the adventitious C 1s peak at 285 eV.

### FT-IR and Raman Spectroscopy Characterization

Raman analyses were performed using a Renishaw inVia apparatus equipped with a Leica microscope with 50 × /20 × /5× objectives and a 785 nm diode laser. System calibration was performed on the 520 cm^–1^ peak of a n-doped silicon wafer (laser power of 5%, acquisition time of 15 s and 4 accumulations).

The infrared spectra were obtained using a Cary 680 Agilent Technologies FTIR spectrometer (Agilent Technologies, Milano, Italy). The measurements were performed in attenuated total reflectance using a Pike Miracle attachment equipped with a ZnSe crystal. ATR-FTIR spectra were collected in the spectral region between 600–1800 cm^–1^, with a resolution of 4 cm^–1^ and averaging 32 scans. All spectra were ATR and baseline corrected.

### High-Performance Liquid Chromatography (HPLC) Analysis of Red and White Wines

HPLC analyses were carried out using a Varian Prostar high-performance liquid chromatography system equipped with dual isocratic pumps and a high-pressure mixing unit, a 20 μL injection loop (Rheodyne, Cotati, CA, USA) and a single-wavelength UV detector (Varian ProStar 325). The separation was achieved using a C18 reverse-phase partition chromatography column (Biobasic-18) with a length of 15 cm, an internal diameter of 2.1 mm, and 3 μm particle size and applying a pressure of 1000 psi (68,95 bar). The system was controlled by a PC running Galaxie workstation software and the column temperature was maintained at 28 °C using a column thermostatting device. A method already known in literature was used for QU separation/detection from the wines. UV–Vis detection was performed at a wavelength of 360 nm. The mobile phases were composed of solvent A (water–acetonitrile–acetic acid, 67:32:1 v/v/v) and solvent B (water–acetic acid, 99:1 v/v). The gradient elution program was as follows: initial conditions at 0 min (20% A, 80% B); 4 min (30% A, 70% B); 8 min (40% A, 60% B); 12 min (65% A, 35% B); 16 min (80% A, 20% B); 20 min (95% A, 5% B); 21.8 min (97% A, 3% B); 24 min (100% A), and held until 30 min. The flow rate was set at 0.5 mL min^–1^, with an injection volume of 20 μL. Stock standard solutions of QU were prepared in methanol and stored at 4 °C in the darkness, then used to construct the calibration curve. Prior to analysis, wine samples were diluted 1:1 (v/v) with methanol.

## Supporting Information Available

Additional details about computational simulations, experimental setup, materials, and methods, including photographs of experimental setup (PDF)

## References

[ref1] Wagner M.; Stanbury P.; Dietrich T.; Döring J.; Ewert J.; Foerster C.; Freund M.; Friedel M.; Kammann C.; Koch M.; Owtram T.; Schultz H. R.; Voss-Fels K.; Hanf J. Developing a Sustainability Vision for the Global Wine Industry. Sustainability 2023, 15 (13), 10487 10.3390/su151310487.

[ref2] Haseeb S.; Alexander B.; Santi R. L.; Liprandi A. S.; Baranchuk A. What’s in Wine? A Clinician’s Perspective. Trends Cardiovasc Med. 2019, 29 (2), 97–106. 10.1016/j.tcm.2018.06.010. 30104174

[ref3] Gutiérrez-Escobar R.; Aliaño-González M. J.; Cantos-Villar E. Wine Polyphenol Content and Its Influence on Wine Quality and Properties: A Review. Molecules 2021, 26 (3), 718 10.3390/molecules26030718. 33573150 PMC7866523

[ref4] Bramley R. G. V.; Ouzman J.; Boss P. K. Variation in Vine Vigour, Grape Yield and Vineyard Soils and Topography as Indicators of Variation in the Chemical Composition of Grapes, Wine and Wine Sensory Attributes. Aust J. Grape Wine Res. 2011, 17 (2), 217–229. 10.1111/j.1755-0238.2011.00136.x.

[ref5] Simonetti G.; Buiarelli F.; Bernardini F.; Di Filippo P.; Riccardi C.; Pomata D. Profile of Free and Conjugated Quercetin Content in Different Italian Wines. Food Chem. 2022, 382, 132377 10.1016/j.foodchem.2022.132377. 35158269

[ref6] Zhang M.; Swarts S. G.; Yin L.; Liu C.; Tian Y.; Cao Y.; Swarts M.; Yang S.; Zhang S. B.; Zhang K.; Ju S.; Olek D. J.; Schwartz L.; Keng P. C.; Howell R.; Zhang L.; Okunieff P. Antioxidant Properties of Quercetin. Adv. Exp. Med. Biol. 2011, 701, 283–289. 10.1007/978-1-4419-7756-4_38. 21445799

[ref7] Jafarinia M.; Sadat Hosseini M.; Kasiri N.; Fazel N.; Fathi F.; Ganjalikhani Hakemi M.; Eskandari N. Quercetin with the Potential Effect on Allergic Diseases. Allergy, Asthma and Clinical Immunology 2020, 16 (1), 1–11. 10.1186/s13223-020-00434-0. PMC7227109 32467711

[ref8] Gambuti A.; Picariello L.; Rinaldi A.; Forino M.; Blaiotta G.; Moine V.; Moio L. New Insights into the Formation of Precipitates of Quercetin in Sangiovese Wines. J. Food Sci. Technol. 2020, 57 (7), 2602–2611. 10.1007/s13197-020-04296-7. 32549610 PMC7270466

[ref9] Wilson A.; Ferrandino A.; Giacosa S.; Novello V.; Guidoni S. The Effect of Temperature and UV Manipulation on Anthocyanins, Flavonols, and Hydroxycinnamoyl-Tartrates in Cv Nebbiolo Grapes (Vitis Vinifera L.). Plants 2024, 13 (22), 3158 10.3390/plants13223158. 39599366 PMC11597326

[ref10] Forzato C.; Vida V.; Berti F. Biosensors and Sensing Systems for Rapid Analysis of Phenolic Compounds from Plants: A Comprehensive Review. Biosensors 2020, 10 (9), 105 10.3390/bios10090105. 32846992 PMC7557957

[ref11] Garcia-Cruz A.; Ahmad O. S.; Alanazi K.; Piletska E.; Piletsky S. A. Generic Sensor Platform Based on Electro-Responsive Molecularly Imprinted Polymer Nanoparticles (e-NanoMIPs). Microsystems & Nanoengineering 2020, 6 (1), 1–9. 10.1038/s41378-020-00193-3. 34567693 PMC8433225

[ref12] Marinangeli A.; Chianella I.; Radicchi E.; Maniglio D.; Bossi A. M. Molecularly Imprinted Polymers Electrochemical Sensing: The Effect of Inhomogeneous Binding Sites on the Measurements. A Comparison between Imprinted Polyaniline versus NanoMIP-Doped Polyaniline Electrodes for the EIS Detection of 17β-Estradiol. ACS Sens. 2024, 9, 4963 10.1021/acssensors.4c01787. 39206707

[ref13] Leibl N.; Haupt K.; Gonzato C.; Duma L. Molecularly Imprinted Polymers for Chemical Sensing: A Tutorial Review. Chemosensors 2021, 9 (6), 123 10.3390/chemosensors9060123.

[ref14] Mazzotta E.; Di Giulio T.; Malitesta C. Electrochemical Sensing of Macromolecules Based on Molecularly Imprinted Polymers: Challenges, Successful Strategies, and Opportunities. Anal. Bioanal. Chem. 2022, 414 (18), 5165–5200. 10.1007/s00216-022-03981-0. 35277740 PMC8916950

[ref15] Gagliani F.; Di Giulio T.; Asif M. I.; Malitesta C.; Mazzotta E. Boosting Electrochemical Sensing Performances Using Molecularly Imprinted Nanoparticles. Biosensors 2024, 14 (7), 358 10.3390/bios14070358. 39056634 PMC11274585

[ref16] Goldoni R.; Thomaz D. V.; Ottolini M.; Di Giulio S.; Di Giulio T. Characterization of In Situ Electrosynthesis of Polyaniline on Pencil Graphite Electrodes through Electrochemical, Spectroscopical and Computational Methods. J. Mater. Sci. 2024, 59 (23), 10287–10308. 10.1007/s10853-024-09745-8.

[ref17] Sellergren B.; Allender C. J. Molecularly Imprinted Polymers: A Bridge to Advanced Drug Delivery. Adv. Drug Deliv Rev. 2005, 57 (12), 1733–1741. 10.1016/j.addr.2005.07.010. 16253386

[ref18] Di Giulio T.; Mazzotta E.; Malitesta C. Molecularly Imprinted Polyscopoletin for the Electrochemical Detection of the Chronic Disease Marker Lysozyme. Biosensors 2020, 11 (1), 3 10.3390/bios11010003. 33374794 PMC7823763

[ref19] Xu L.; Pan M.; Fang G.; Wang S. Carbon Dots Embedded Metal-Organic Framework@molecularly Imprinted Nanoparticles for Highly Sensitive and Selective Detection of Quercetin. Sens Actuators B Chem. 2019, 286, 321–327. 10.1016/j.snb.2019.01.156.

[ref20] Hu Y.; Feng T.; Li G. A Novel Solid Fluorescence Method for the Fast Determination of Quercetin in Biological Samples Based on the Quercetin–Al(III) Complex Imprinted Polymer. Spectrochim Acta A Mol. Biomol Spectrosc 2014, 118, 921–928. 10.1016/j.saa.2013.09.076. 24161857

[ref21] Mantashloo R.; Bahar S. Synthesis of Magnetic Graphene Quantum Dots Based Molecularly Imprinted Polymers for Fluorescent Determination of Quercetin. Microchemical Journal 2023, 185, 108233 10.1016/j.microc.2022.108233.

[ref22] Ganjeh A. A.; Arvand M.; Habibi M. F. Electrostatically Self-Assembled Magnetized MXene/Copper Ferrite Nanospheres Hybrids: Evaluation of Molecularly Imprinted Electrochemical Sensor for the Quercetin Antioxidant Supplement. Microchemical Journal 2024, 207, 112026 10.1016/j.microc.2024.112026.

[ref23] Bandyopadhyay D.; Nag S.; Das D.; Roy R. B. A Novel RGO-Decorated Molecularly Imprinted Polyacrylic Acid Graphite Electrode for the Detection of Quercetin in Food. IEEE Trans Instrum Meas 2024, 73, 1–8. 10.1109/TIM.2024.3398119.

[ref24] Sun S.; Zhang M.; Li Y.; He X. A Molecularly Imprinted Polymer with Incorporated Graphene Oxide for Electrochemical Determination of Quercetin. Sensors 2013, 13 (5), 5493–5506. 10.3390/s130505493. 23698263 PMC3690011

[ref25] Hurkul M. M.; Cetinkaya A.; Yayla S.; Kaya S. I.; Budak F.; Tok K. C.; Gumustas M.; Uzun L.; Ozkan S. A. Highly Selective and Sensitive Molecularly Imprinted Sensors for the Electrochemical Assay of Quercetin in Methanol Extracts of Rubus Sanctus and Fragaria Vesca. Talanta 2024, 273, 125883 10.1016/j.talanta.2024.125883. 38521023

[ref26] Lowdon J. W.; Diliën H.; Singla P.; Peeters M.; Cleij T. J.; van Grinsven B.; Eersels K. MIPs for Commercial Application in Low-Cost Sensors and Assays – An Overview of the Current Status Quo. Sens Actuators B Chem. 2020, 325, 128973 10.1016/j.snb.2020.128973. 33012991 PMC7525251

[ref27] Niu J.; Du M.; Wu W.; Yang J.; Chen Q. Advances in the Selection of Functional Monomers for Molecularly Imprinted Polymers: A Review. J. Sep Sci. 2024, 47 (16), 2400353 10.1002/jssc.202400353. 39164908

[ref28] Dabrowski M.; Lach P.; Cieplak M.; Kutner W. Nanostructured Molecularly Imprinted Polymers for Protein Chemosensing. Biosens Bioelectron 2018, 102, 17–26. 10.1016/j.bios.2017.10.045. 29101784

[ref29] Mazzotta E.; Di Giulio T.; Mariani S.; Corsi M.; Malitesta C.; Barillaro G. Vapor-Phase Synthesis of Molecularly Imprinted Polymers on Nanostructured Materials at Room-Temperature. Small 2023, 19 (38), 2302274 10.1002/smll.202302274. 37222612

[ref30] Antunez E. E.; Martin M. A.; Voelcker N. H. Porous Silicon-Based Sensors for Protein Detection. In Porous Silicon for Biomedical Applications; Elsevier, 2021; pp 359–395. 10.1016/B978-0-12-821677-4.00001-X.

[ref31] De Stefano L.; Rotiroti L.; Rendina I.; Moretti L.; Scognamiglio V.; Rossi M.; D’Auria S. Porous Silicon-Based Optical Microsensor for the Detection of l-Glutamine. Biosens Bioelectron 2006, 21 (8), 1664–1667. 10.1016/j.bios.2005.08.012. 16207529

[ref32] Ruminski A. M.; King B. H.; Salonen J.; Snyder J. L.; Sailor M. J. Porous Silicon-Based Optical Microsensors for Volatile Organic Analytes: Effect of Surface Chemistry on Stability and Specificity. Adv. Funct Mater. 2010, 20 (17), 2874–2883. 10.1002/adfm.201000575.

[ref33] Sailor M. J.; Wu E. C. Photoluminescence-Based Sensing With Porous Silicon Films, Microparticles, and Nanoparticles. Adv. Funct Mater. 2009, 19 (20), 3195–3208. 10.1002/adfm.200900535.

[ref34] Li Y. Y.; Cunin F.; Link J. R.; Gao T.; Betts R. E.; Reiver S. H.; Chin V.; Bhatia S. N.; Sailor M. J. Polymer Replicas of Photonic Porous Silicon for Sensing and Drug Delivery Applications. Science 2003, 299 (5615), 2045–2047. 10.1126/science.1081298. 12663921

[ref35] Mariani S.; Strambini L. M.; Barillaro G. Electrical Double Layer-Induced Ion Surface Accumulation for Ultrasensitive Refractive Index Sensing with Nanostructured Porous Silicon Interferometers. ACS Sens 2018, 3 (3), 595–605. 10.1021/acssensors.7b00650. 29299931

[ref36] Mariani S.; Paghi A.; La Mattina A. A.; Debrassi A.; Dähne L.; Barillaro G. Decoration of Porous Silicon with Gold Nanoparticles via Layer-by-Layer Nanoassembly for Interferometric and Hybrid Photonic/Plasmonic (Bio)Sensing. ACS Appl. Mater. Interfaces 2019, 11 (46), 43731–43740. 10.1021/acsami.9b15737. 31644268

[ref37] Nocerino V.; Rea I.; Siciliano G.; De Stefano L.; Primiceri E. Polymers Modified Porous Silicon Optical (Bio)Sensors. TrAC 2024, 177, 117811 10.1016/j.trac.2024.117811. PMC12109668 40422059

[ref38] Awawdeh K.; Buttkewitz M. A.; Bahnemann J.; Segal E. Enhancing the Performance of Porous Silicon Biosensors: The Interplay of Nanostructure Design and Microfluidic Integration. Microsystems & Nanoengineering 2024, 10 (1), 1–14. 10.1038/s41378-024-00738-w. 39021530 PMC11252414

[ref39] Nicholls I. A.; Golker K.; Olsson G. D.; Suriyanarayanan S.; Wiklander J. G. The Use of Computational Methods for the Development of Molecularly Imprinted Polymers. Polymers 2021, 13 (17), 2841 10.3390/polym13172841. 34502881 PMC8434026

[ref40] Rajpal S.; Mishra P.; Mizaikoff B. Rational In Silico Design of Molecularly Imprinted Polymers: Current Challenges and Future Potential. I. J. Mol. Sci. 2023, 24 (7), 6785 10.3390/ijms24076785. PMC10095314 37047758

[ref41] Ndunda E. N. Molecularly Imprinted Polymers—A Closer Look at the Control Polymer Used in Determining the Imprinting Effect: A Mini Review. J. Mol. Recog. 2020, 33 (11), e2855 10.1002/jmr.2855. 32529728

[ref42] Adeleke V. T.; Ebenezer O.; Lasich M.; Mugo S. M. Theoretical Insights into the Compatibility of Template-Monomer-Crosslinker-Solvent for Cortisol Molecularly Imprinted Polymer Pre-Polymerization. Mol. Syst. Des Eng. 2024, 9 (1), 99–111. 10.1039/D3ME00077J.

[ref43] Guan S.; Wang Y.; Hu T.; Che L.; Wang X.; Huang Y.; Xia Z. Study on the Selectivity of Molecular Imprinting Materials Determined through Hydrogen Bonding on Template Molecular Structures of Flavonoids. Molecules 2024, 29 (6), 1292 10.3390/molecules29061292. 38542926 PMC10975109

[ref44] Das R. S.; Kumar A.; Gaharwar S. S.; Senapati S. K.; Mandavgane S. A. DFT Simulated Quercetin Imprinted Polymer: Selective Recovery of Quercetin from Onion Solid Waste. J. Chromatogr A 2024, 1730, 465151 10.1016/j.chroma.2024.465151. 39002509

[ref45] Majoul N.; Aouida S.; Bessaïs B. Progress of Porous Silicon APTES-Functionalization by FTIR Investigations. Appl. Surf. Sci. 2015, 331, 388–391. 10.1016/j.apsusc.2015.01.107.

[ref46] Orbay S.; Kocaturk O.; Sanyal R.; Sanyal A. Molecularly Imprinted Polymer-Coated Inorganic Nanoparticles: Fabrication and Biomedical Applications. Micromachines 2022, 13 (9), 1464 10.3390/mi13091464. 36144087 PMC9501141

[ref47] Osojnik Črnivec I. G.; Skrt M.; Polak T.; Šeremet D.; Mrak P.; Komes D.; Vrhovšek U.; Poklar Ulrih N. Aspects of Quercetin Stability and Its Liposomal Enhancement in Yellow Onion Skin Extracts. Food Chem. 2024, 459, 140347 10.1016/j.foodchem.2024.140347. 38991436

[ref48] Valentino M.; Imbriano A.; Tricase A.; Della Pelle F.; Compagnone D.; Macchia E.; Torsi L.; Bollella P.; Ditaranto N. Electropolymerized Molecularly Imprinted Polypyrrole Film for Dimethoate Sensing: Investigation on Template Removal after the Imprinting Process. Analytical Methods 2023, 15 (10), 1250–1253. 10.1039/D2AY02024F. 36861684

[ref49] Zhang J.; Gai M.; Ignatov A. V.; Dyakov S. A.; Wang J.; Gippius N. A.; Frueh J.; Sukhorukov G. B. Stimuli-Responsive Microarray Films for Real-Time Sensing of Surrounding Media, Temperature, and Solution Properties via Diffraction Patterns. ACS Appl. Mater. Interfaces 2020, 12 (16), 19080–19091. 10.1021/acsami.0c05349. 32223175

[ref50] Bourhis K.; Blanc S.; Mathe C.; Dupin J. C.; Vieillescazes C. Spectroscopic and Chromatographic Analysis of Yellow Flavonoidic Lakes: Quercetin Chromophore. Appl. Clay Sci. 2011, 53 (4), 598–607. 10.1016/j.clay.2011.05.009.

[ref51] Longo L.; Vasapollo G.; Guascito M. R.; Malitesta C. New Insights from X-Ray Photoelectron Spectroscopy into the Chemistry of Covalent Enzyme Immobilization, with Glutamate Dehydrogenase (GDH) on Silicon Dioxide as an Example. Anal Bioanal Chem. 2006, 385 (1), 146–152. 10.1007/s00216-006-0398-1. 16583206

[ref52] San Andrés E.; Del Prado A.; Martínez F. L.; Mártil I.; Bravo D.; López F. J. Rapid Thermal Annealing Effects on the Structural Properties and Density of Defects in SiO2 and SiNx:H Films Deposited by Electron Cyclotron Resonance. J. Appl. Phys. 2000, 87 (3), 1187–1192. 10.1063/1.371996.

[ref53] Kulkarni G.; Kandesar P.; Velhal N.; Kim H.; Puri V. Facile Synthesis of Coral Cauliflower-like Polypyrrole Hemispheres toward Screening Electromagnetic Interference Pollution. J. Appl. Polym. Sci. 2021, 138 (22), 50447 10.1002/app.50447.

[ref54] Bang J. H.; Choi M. S.; Mirzaei A.; Oum W.; Han S.; Kim S. S.; Kim H. W. Porous Si/SnO2 Nanowires Heterostructures for H2S Gas Sensing. Ceram. Int. 2020, 46 (1), 604–611. 10.1016/j.ceramint.2019.09.010.

[ref55] Shahryari Z.; Gheisari K.; Yeganeh M.; Ramezanzadeh B. Corrosion Mitigation Ability of Differently Synthesized Polypyrrole (PPy-FeCl3 & PPy-APS) Conductive Polymers Modified with Na2MoO4 on Mild Steel in 3.5% NaCl Solution: Comparative Study and Optimization. Corros. Sci. 2021, 193, 109894 10.1016/j.corsci.2021.109894.

[ref56] Santos M. J. L.; Brolo A. G.; Girotto E. M. Study of Polaron and Bipolaron States in Polypyrrole by in Situ Raman Spectroelectrochemistry. Electrochim. Acta 2007, 52 (20), 6141–6145. 10.1016/j.electacta.2007.03.070.

[ref57] Li M.; Wei Z.; Jiang L. Polypyrrole Nanofiber Arrays Synthesized by a Biphasic Electrochemical Strategy. J. Mater. Chem. 2008, 18 (19), 2276–2280. 10.1039/b800289d.

[ref58] Teslova T.; Corredor C.; Livingstone R.; Spataru T.; Birke R. L.; Lombardi J. R.; Cañamares M. V.; Leona M. Raman and Surface-Enhanced Raman Spectra of Flavone and Several Hydroxy Derivatives. J. Raman Spectrosc. 2007, 38 (7), 802–818. 10.1002/jrs.1695.

[ref59] Sato S.; Numata Y. Simultaneous Quantitative Analysis of Quercetin and Rutin in Tartary Buckwheat Flour by Raman Spectroscopy and Partial Least Square Regression. J.F. Comp. Anal. 2024, 128, 105991 10.1016/j.jfca.2024.105991.

[ref60] Gutiérrez-Escobar R.; Aliaño-González M. J.; Cantos-Villar E. Wine Polyphenol Content and Its Influence on Wine Quality and Properties: A Review. Molecules 2021, 26 (3), 718 10.3390/molecules26030718. 33573150 PMC7866523

[ref61] García-Calzón J. A.; Díaz-García M. E. Characterization of Binding Sites in Molecularly Imprinted Polymers. Sens Actuators B Chem. 2007, 123 (2), 1180–1194. 10.1016/j.snb.2006.10.068.

[ref62] Vuorinen H.; Määttä K.; Törrönen R. Content of the Flavonols Myricetin, Quercetin, and Kaempferol in Finnish Berry Wines. J. Agric. Food Chem. 2000, 48 (7), 2675–2680. 10.1021/jf991388o. 11032478

[ref63] Fabjanowicz M.; Płotka-Wasylka J.; Namieśnik J. Detection, Identification and Determination of Resveratrol in Wine. Problems and Challenges. TrAC 2018, 103, 21–33. 10.1016/j.trac.2018.03.006.

[ref64] Karrat A.; Palacios-Santander J. M.; Amine A.; Cubillana-Aguilera L. A Novel Magnetic Molecularly Imprinted Polymer for Selective Extraction and Determination of Quercetin in Plant Samples. Anal. Chim. Acta 2022, 1203, 339709 10.1016/j.aca.2022.339709. 35361431

[ref65] Mills D. A.; Phister T.; Neeley E.; Johannsen E. Wine Fermentation. Molecular Techniques in the Microbial Ecology of Fermented Foods 2008, 162–192. 10.1007/978-0-387-74520-6_6.

[ref66] de Quirós A. R. B.; Lage-Yusty M. A.; López-Hernández J. HPLC-Analysis of Polyphenolic Compounds in Spanish White Wines and Determination of Their Antioxidant Activity by Radical Scavenging Assay. Food Research International 2009, 42 (8), 1018–1022. 10.1016/j.foodres.2009.04.009.

[ref67] Qiu H.; Luo C.; Sun M.; Lu F.; Fan L.; Li X. A Novel Chemiluminescence Sensor for Determination of Quercetin Based on Molecularly Imprinted Polymeric Microspheres. Food Chem. 2012, 134 (1), 469–473. 10.1016/j.foodchem.2012.02.102.

[ref68] Bandyopadhyay D.; Nag S.; Das D.; Roy R. B. A Novel RGO-Decorated Molecularly Imprinted Polyacrylic Acid Graphite Electrode for the Detection of Quercetin in Food. IEEE Trans Instrum Meas 2024, 73, 1–8. 10.1109/TIM.2024.3398119.

[ref69] Weigend F.; Furche F.; Ahlrichs R. Gaussian Basis Sets of Quadruple Zeta Valence Quality for Atoms H–Kr. J. Chem. Phys. 2003, 119 (24), 12753–12762. 10.1063/1.1627293.

[ref70] Mariani S.; Robbiano V.; Strambini L. M.; Debrassi A.; Egri G.; Dähne L.; Barillaro G. Layer-by-Layer Biofunctionalization of Nanostructured Porous Silicon for High-Sensitivity and High-Selectivity Label-Free Affinity Biosensing. Nat. Commun. 2018, 9 (1), 5256 10.1038/s41467-018-07723-8. 30531860 PMC6288083

