# Supplementary material for: A Molecularly Imprinted Polymer-Based Porous Silicon Optical Sensor for Quercetin Detection in Wines
Source: ACS Appl Mater Interfaces. 2025 Feb 11;17(8):12663–75. doi: 10.1021/acsami.4c21238 (PMC11873946; doi:10.1021/acsami.4c21238)
Supplement: Supplementary file 1 — am4c21238_si_001.pdf [file am4c21238_si_001.pdf]

SUPPORTING INFORMATION

A Molecularly Imprinted Polymer-based Porous Silicon Optical Sensor for Quercetin Detection in Wines

Tiziano Di Giulio[a,§], Ibrar Muhammad Asif[a,§], Martina Corsi[b], Soumya Rajpal[c], Boris Mizaikoff[c, d], Nicoletta Ditaranto[e], Giuseppe E. De Benedetto[f], Cosimino Malitesta[a], Giuseppe Barillaro[b,*], Elisabetta Mazzotta[a,*]

[a] Laboratory of Analytical Chemistry, Department of Biological and Environmental Sciences and Technologies (Di.S.Te.B.A.), University of Salento, via Monteroni, 73100 Lecce, Italy

[b] Information Engineering Department, University of Pisa, via G. Caruso 16, 56122 Pisa, Italy

[c] Institute of Analytical and Bioanalytical Chemistry, Ulm University, Albert-Einstein-Allee 11, 89081 Ulm, Germany

[d] Hahn-Schickard, Sedanstrasse 14, 89077 Ulm

[e] Chemistry Department, Aldo Moro University of Bari, Via Orabona 4, 70126 Bari, Italy

[f] Laboratory of Analytical Mass Spectrometry, Cultural Heritage Department, University of Salento, Via Monteroni, 73100 Lecce, Italy

E-mail: elisabetta.mazzotta@unisalento.it, giuseppe.barillaro@unipi.it

[§] T.D.G and M.I.A. contributed equally to this work

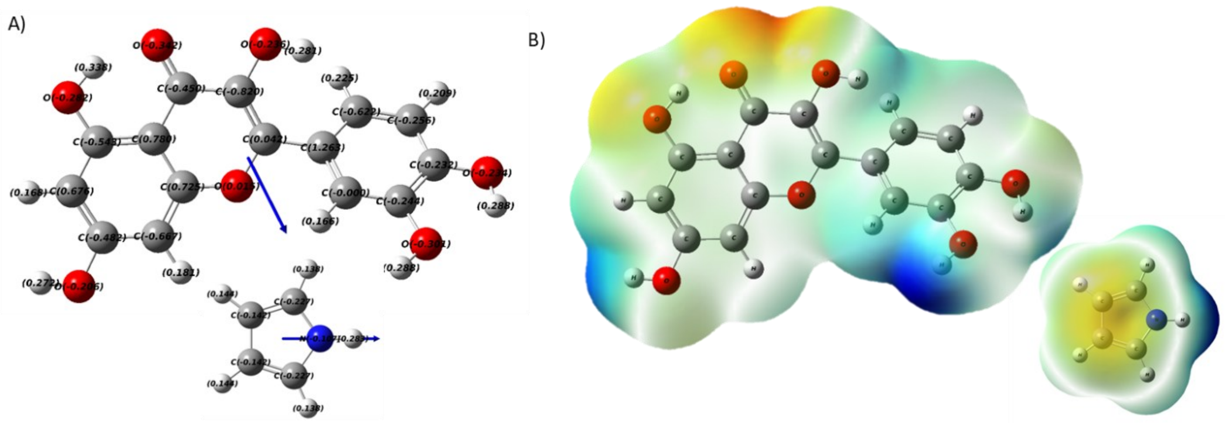

Figure S1. QU and Py molecules showing Mulliken charges (A) and molecular electrostatic potential mapping (B). The colour denotes the reactivity of the region; blue is most positive electrostatic potential, red is most electronegative electrostatic potential and green is for neutral potential.

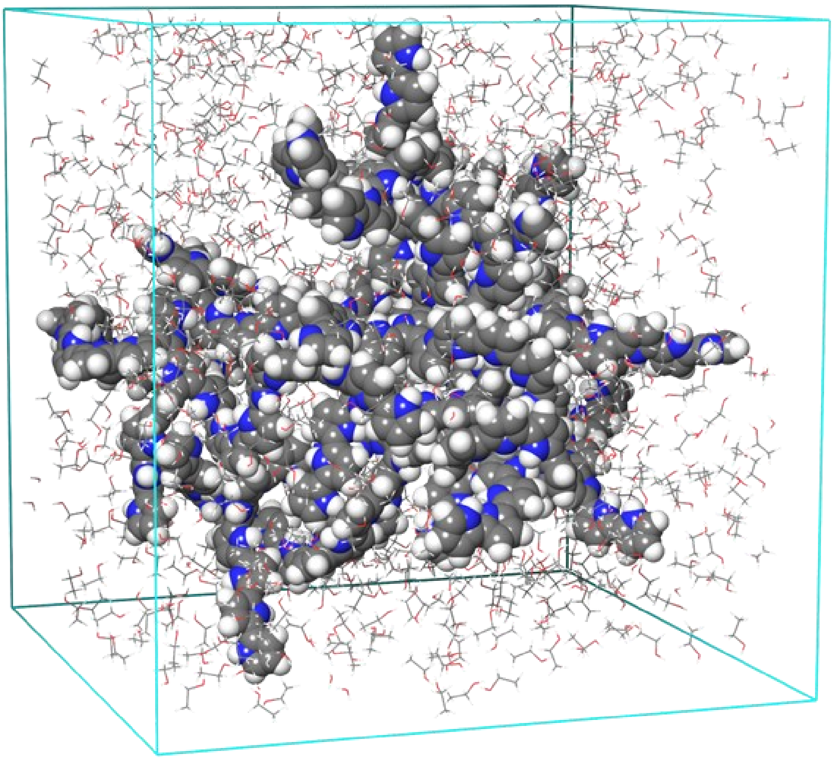

Figure S2. 3D representation of an amorphous polypyrrole (PPy) matrix in CPK representation, surrounded by solvent molecules (water-ethanol, 4:1) in wireframe format, highlighting the polymer-solvent system within the simulated box.

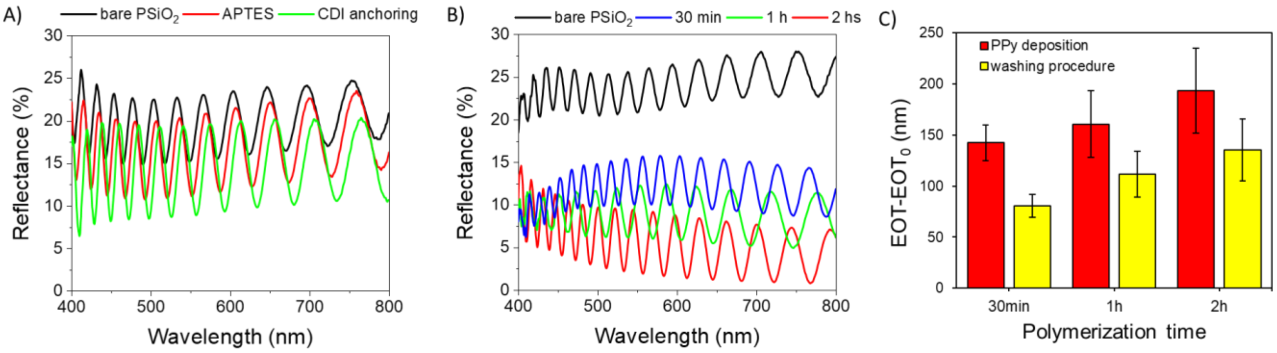

Figure S3. A) Reflectance spectra recorded in air on a $PSiO_2$ scaffold during different functionalization steps: bare $PSiO_2$ (black line), silanization with APTES (red line) and $PSiO_2$ exposure to CDI (green line). B) Reflectance spectra recorded in air on a $PSiO_2$ scaffold after polymer deposition using different polymerization times: 30 min (blue line), 1h (green line) and 2 hs (red line). C) Effective optical thickness changes ($EOT-EOT_0$) achieved after polymer deposition for different time periods, namely 30 min, 1 h and 2 hs and after the washing procedures to obtain the MIPs; the EOT value recorded after quercetin anchoring on $PSiO_2$ scaffold (EOT_0) is used as reference (n=3 samples). Data are presented as mean (± s.d).

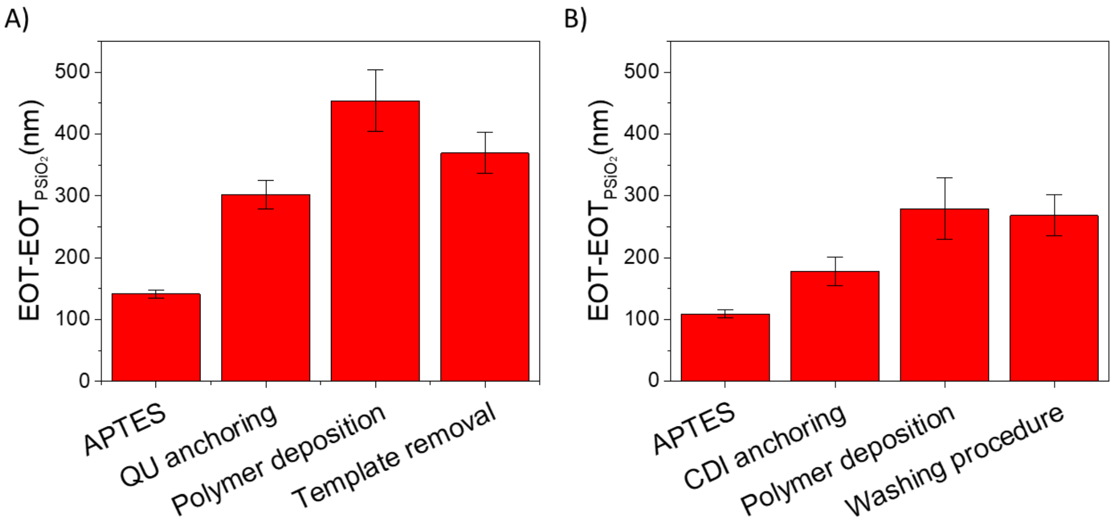

Figure S4. Effective optical thickness changes (EOT-EOT_{PSiO2}) achieved for each functionalization step of A) MIP- and B) NIP-sensors; the EOT value of bare $PSiO_2$ (EOT_{PSiO2}) scaffold is used as reference (n=3 samples). All data are presented as mean (± s.d).

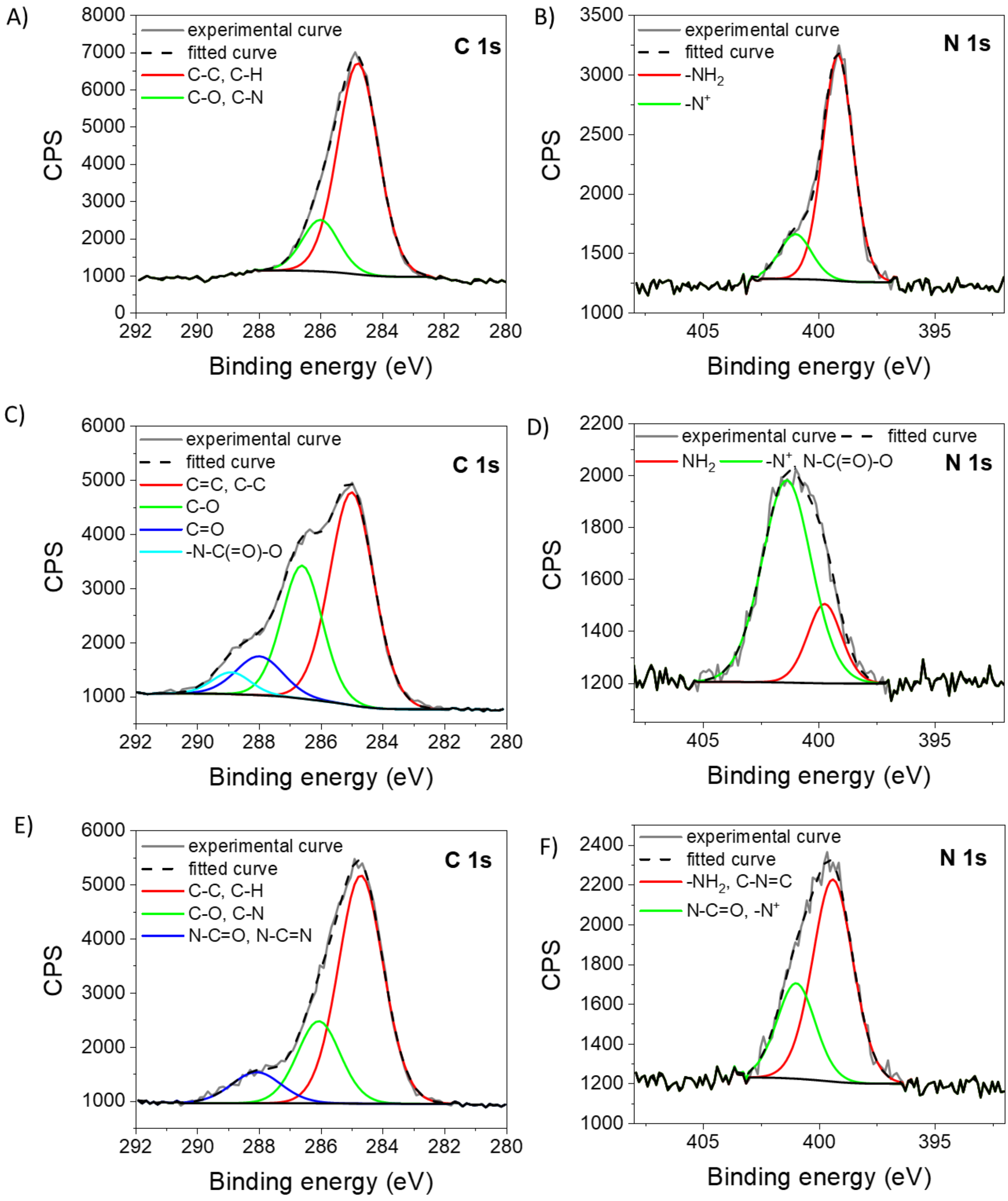

Figure S5. Detailed C 1s signals recorded after A) $PSiO_2$ functionalization with APTES, C) quercetin anchoring and E) $PSiO_2$ scaffolds exposed to CDI. High-definition N 1s spectra recorded after B) $PSiO_2$ functionalization with APTES, D) quercetin anchoring and F) $PSiO_2$ scaffolds exposed to CDI. Spectra are fitted and charging corrected.

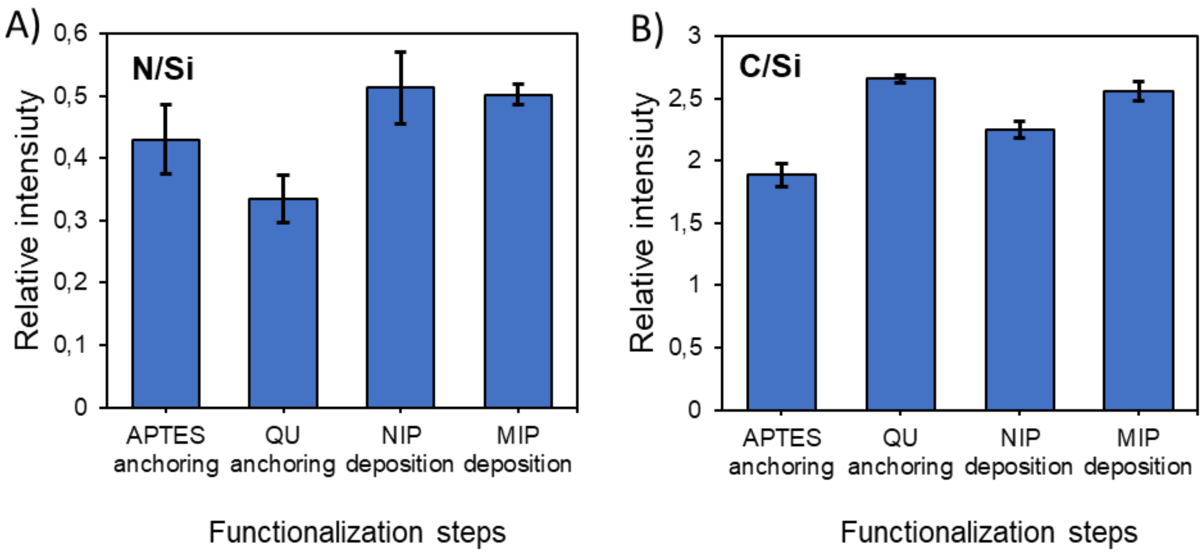

Figure S6. A) N/Si and B) C/Si atomic ratio calculated from XPS analysis of $PSiO_2$ scaffold after each functionalization step up to NIP/MIP deposition. For all the samples, three different measurement positions were analyzed (n = 3). Data are presented as mean (±s.d.).

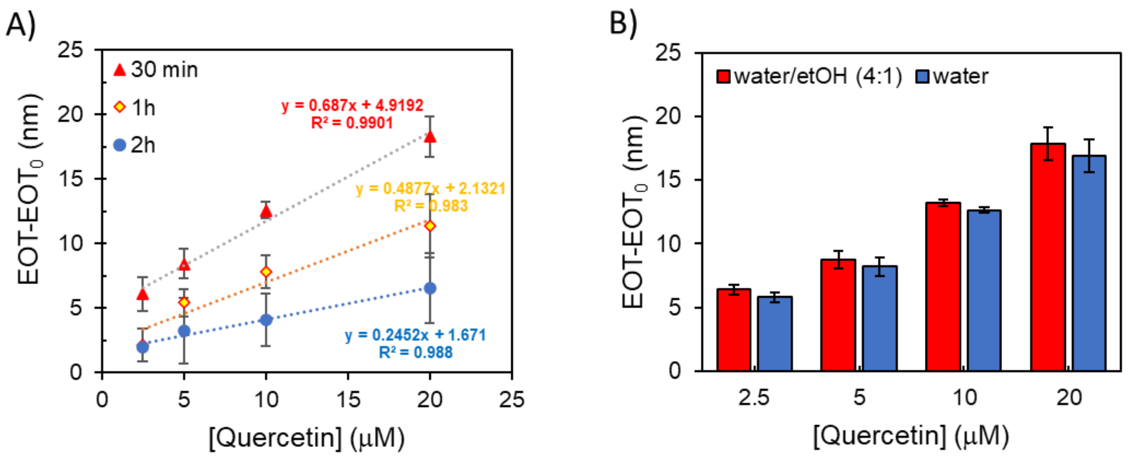

Figure S7. A) Calibration curves (EOT-EOT_0 vs quercetin concentration) recorded on MIP-sensors prepared using different deposition times, namely 30 minutes, 1 hour and 2 hours. The sensors were used in quercetin detection tests using standard solutions prepared in water (from 2.5 to 20 µM). EOT_0 is measured in buffer solution without quercetin and used as reference (n=3 samples). Data are presented as mean (± s.d). B) Comparison of responses (EOT-EOT_0 vs quercetin concentration) of MIP-sensors, obtained using a deposition time of 30 minutes, to quercetin solutions prepared in ultra-pure water (blue column) and water/EtOH (4:1, v/v) mixture (red columns). EOT_0 is measured in buffer solution without quercetin and used as reference (n=3 samples). Data are presented as mean (± s.d).

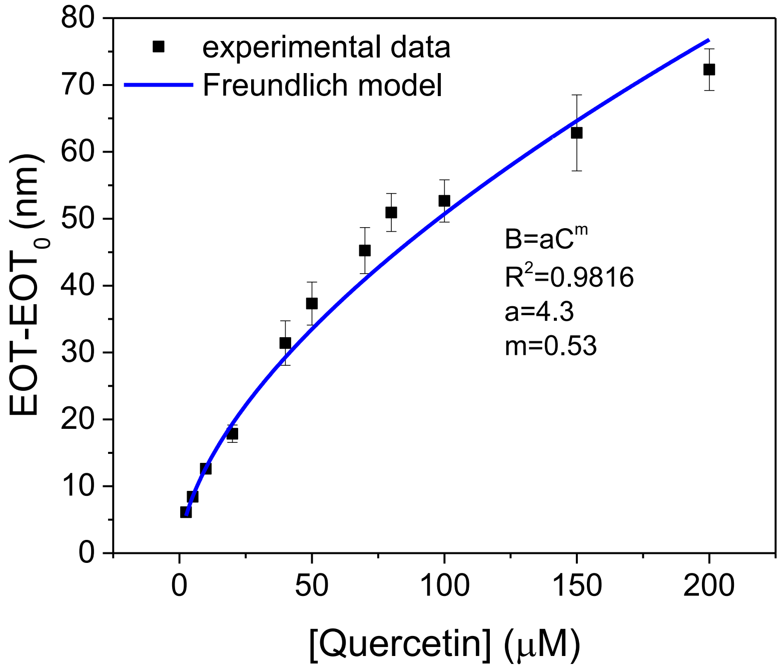

Figure S8. Best-fitting of the MIP sensor calibration curve (black dots) using the Freundlich isotherm model (blue trace), where B = EOT-EOT$_0$ is the sensor output and C is quercetin concentration in solution. Fitting parameters are a=4.3 related to the median binding affinity and m=0.53, the heterogeneity index. Experimental data are presented as the mean value of n = 3 samples, with error bars representing the standard deviation.

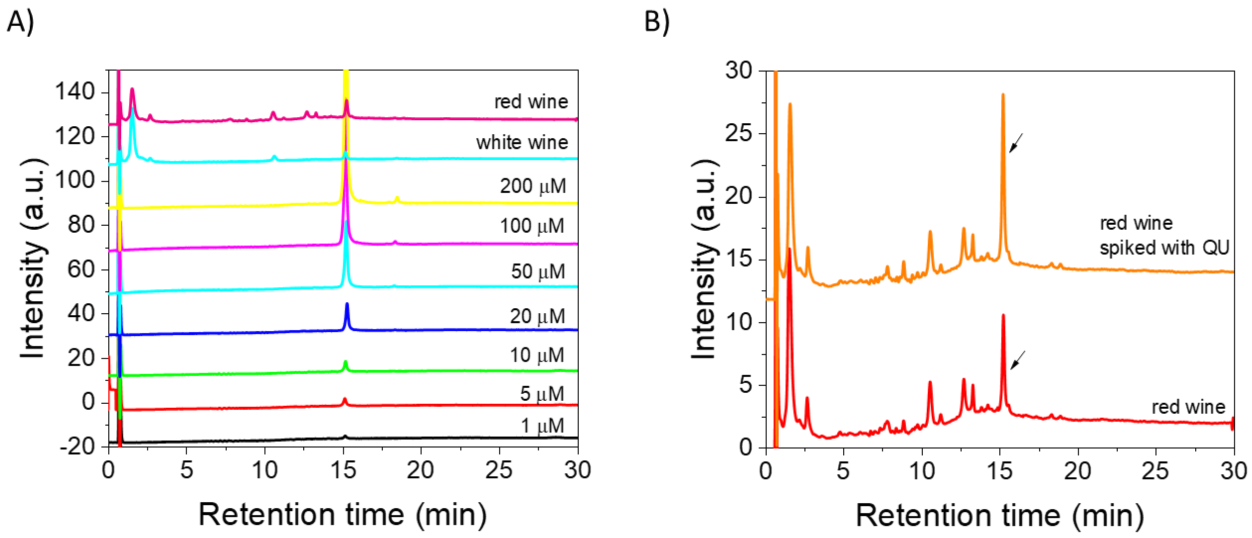

Figure S9. A) HPLC-chromatograms recorded for quercetin standard solutions (from 1 to 200 μM) and for white and red wines from Salento region. Quercetin standard solutions were prepared in methanol. White and red wines were diluted in methanol (1:1, v/v) before their analysis. Quercetin has a retention time of 15.2 min. B) HPLC-chromatograms recorded for red wine samples. As control the red wine used was spiked with QU (solution prepared in methanol), giving an increase of quercetin peak at 15.2 min (indicated by an arrow).
